# Spatial distribution of marine airborne bacterial communities

**DOI:** 10.1002/mbo3.253

**Published:** 2015-03-19

**Authors:** Jasmin S Seifried, Antje Wichels, Gunnar Gerdts

**Affiliations:** Department of Microbial Ecology, Biologische Anstalt Helgoland, Alfred Wegener Institute Helmholtz Centre for Polar and Marine ResearchHelgoland, Germany

**Keywords:** Bioaerosols, impinger, marine, pyrosequencing, trajectories

## Abstract

The spatial distribution of bacterial populations in marine bioaerosol samples was investigated during a cruise from the North Sea to the Baltic Sea via Skagerrak and Kattegat. The analysis of the sampled bacterial communities with a pyrosequencing approach revealed that the most abundant phyla were represented by the Proteobacteria (49.3%), Bacteroidetes (22.9%), Actinobacteria (16.3%), and Firmicutes (8.3%). Cyanobacteria were assigned to 1.5% of all bacterial reads. A core of 37 bacterial OTUs made up more than 75% of all bacterial sequences. The most abundant OTU was *Sphingomonas* sp. which comprised 17% of all bacterial sequences. The most abundant bacterial genera were attributed to distinctly different areas of origin, suggesting highly heterogeneous sources for bioaerosols of marine and coastal environments. Furthermore, the bacterial community was clearly affected by two environmental parameters – temperature as a function of wind direction and the sampling location itself. However, a comparison of the wind directions during the sampling and calculated backward trajectories underlined the need for more detailed information on environmental parameters for bioaerosol investigations. The current findings support the assumption of a bacterial core community in the atmosphere. They may be emitted from strong aerosolizing sources, probably being mixed and dispersed over long distances.

## Introduction

The presence of bacteria in the atmosphere and their relevance for atmospheric processes have been the subject of investigations since the 19th century (see Després et al. 2012 and references therein). Although inorganic particles such as sulfate, sea salt, mineral dust, and volcanic ashes are comparably well studied, biological particles received only little attention which is also due to a former underestimation of their occurrence in the atmosphere (see Matthias-Maser and Jaenicke 1995; Jaenicke 2005; Després et al. 2012 and references therein). Primary biological aerosol particles (PBAP) are defined as solid airborne particles originating from biological organisms including microorganisms, plant debris, pollen, fungi spores, and cells as well as plant wax released by living organisms (IGAP 1992). Their release into the atmosphere can be active and/or passive, emitting from nearly all kinds of surfaces. Marine PBAP mainly emerge from a bubble-bursting process (Blanchard and Woodcock 1980; Blanchard and Syzdek 1982) by which small air bubbles (formed by breaking waves/whitecaps) get suspended to the depth of several meters into the water column. While resurfacing, small particles get collected and accumulated on the bubbles' surfaces with an enrichment factor of 15–25 (Aller et al. 2005), thus exceeding the natural concentration of bacteria in the surrounding waters. When the bubbles reach the water surface, collected material is ejected into the atmosphere. The same process creates the concentration of sea salt in marine air (Burrows et al. 2009). As oceans cover more than 70% of the global surface with a bacterial concentration of 3 × 10^5^–5 × 10^6^ cm^−3^ in surface waters, it is assumed that many PBAP are emitted from the oceans. Nevertheless, still little is known about bioaerosols from marine environments. Most PBAP studies focused on public health concerns in hospitals and workplaces (e.g., Eames et al. 2009; Tang 2009; D'Arcy et al. 2012; Eduard et al. 2012), and the few outdoor studies were mainly conducted in urban and rural environments (e.g., Maron et al. 2005; Després et al. 2007; Li et al. 2010). To date, the lack of standardized sampling methods and/or analyses in PBAP investigations remains one of the main problems in bioaerosol investigation (Burrows et al. 2009; Després et al. 2012; Gandolfi et al. 2013). Interpretation and comparison of findings from different studies are, thus, impeded by the employment of different sampling techniques.

Despite their inevitable biases (selectivity of media, incubation time, incubation temperature), most investigations on PBAPs used culture-dependent techniques. This preselection for special types of bacteria is a considerable disadvantage for investigations on bioaerosols as their origin can be strongly heterogeneous. Recent studies showed that culture-dependent and culture-independent methods produce different results for the same bacterial communities (Cho and Hwang 2011; Ravva et al. 2012), although some findings may overlap (Fahlgren et al. 2010; Urbano et al. 2011). Furthermore, even if viable, only a small fraction of environmental bacteria are culturable (around 0.001–0.01% of seawater bacteria; Colwell 2000) and culturability rapidly declines after aerosolization (Heidelberg et al. 1997). Studies on marine/coastal bioaerosols, so far, used a combination of culture-dependent and culture-independent methods. Bacteria were identified by cloning (Fahlgren et al. 2010; Urbano et al. 2011) or Denaturing Gradient Gel Electrophoresis (DGGE) with subsequently conducted sequencing of bands (Cho and Hwang 2011). Both resulted in only few sequences which most likely reflected only the most abundant taxa. As these methods do not allow for covering the bacterial communities as a whole, the current study employed a high-throughput pyrosequencing approach which has been successfully used before (Bowers et al. 2009, 2011a). Pyrosequencing provides a detailed description of the microbial community including rare taxa and facilitates robust statistical analyses.

Considering the advantages of pyrosequencing, the current study aimed to characterize the bacterial community of marine bioaerosols without the restrictions of culture-dependent approaches and to test for the influences of spatial heterogeneity (different sampling locations with different surrounding ecosystems) and selected environmental parameters which may influence the bacterial airborne community (e.g., wind direction, rain, or temperature).

## Material and Methods

### Sample collection

**Figure 1:**
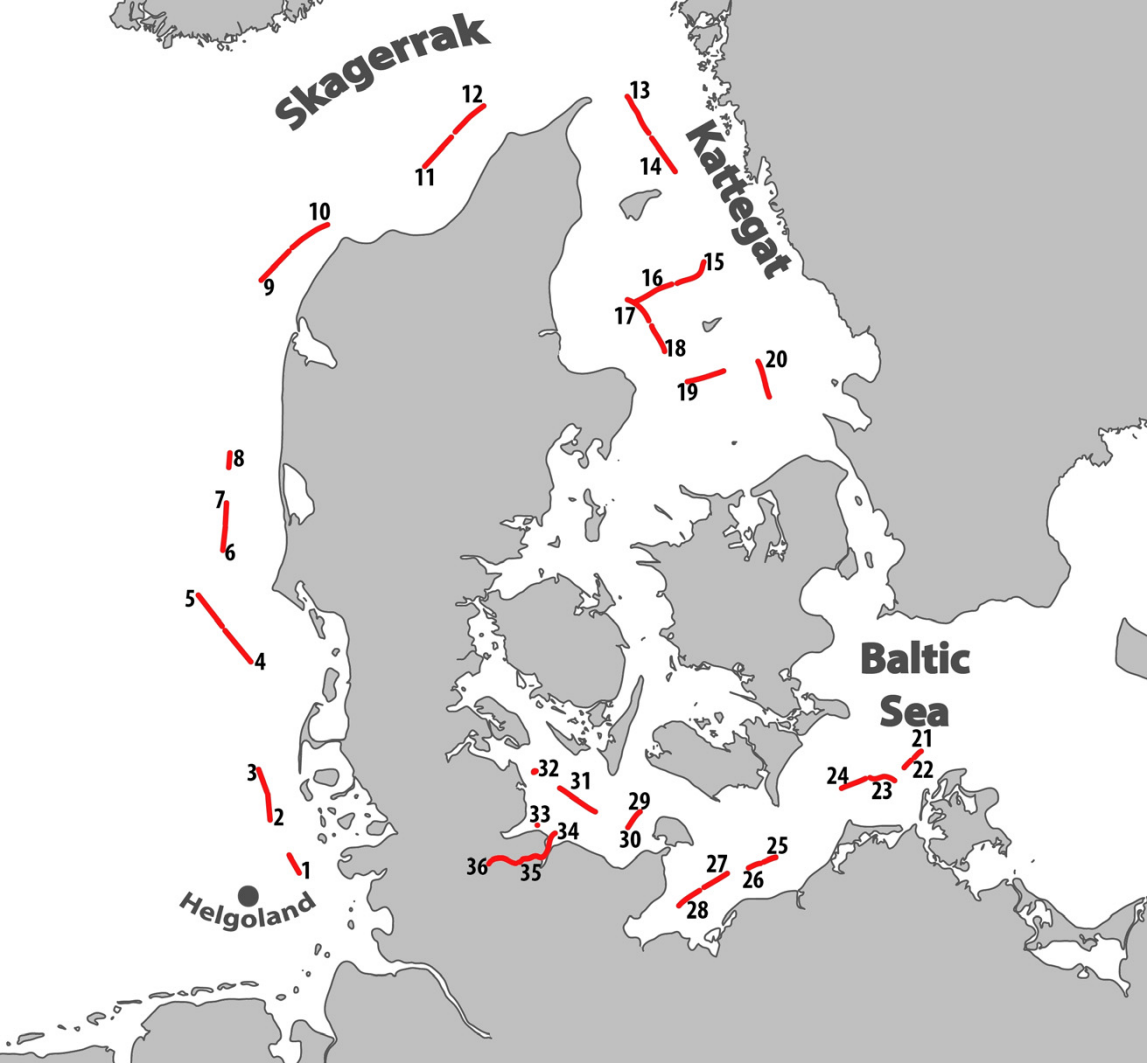
Sampling transects in chronological order, conducted in August 2011. Each transect represents the total sampling time of 1 h.

Bioaerosol samples were collected in August 2011 during a ship cruise from the North Sea to the Baltic Sea via Kattegat and Skagerrak (Fig.1). The samples were only collected when the ship was moving in order to eliminate possible biases which could be introduced by the ship chimney. In order to cover a wide spatial range in distribution, samples were collected in between stations – two in the morning and two in the afternoon. An impingement aerosol sampler (Dycor XMX/2L MIL; Edmonton, Canada) was employed to gather the total number of 36 samples. Before each sampling, the sampling device was cleaned with 70% ethanol. The impingement aerosol sampler was positioned on the top deck of the research vessel, 10 m above sea level (a.s.l.). This sampling device is qualified for processing high volumes of air, stripping away larger dust particles and accumulating the very small micro debris and aerosols within a diameter of 1–10 *μ*m. The airstream is directed into the sterile collection vial through a liquid, which is responsible to wash out particles which are transported with the airstream. As a collection medium, 5 mL of sterile phosphate-buffered saline was used to prevent bacterial cells from bursting. Each sample was taken for an hour consisting of six subsamples (10 min sampling time each) which were pooled for further analysis. In total, 0.72 m^3^ air per hour and sample were collected. The sample was then filtered through 0.2 *μ*m Isopore™ membrane filters (GTTP-type, diameter 13 mm; Millipore, Eschborn, Germany) and stored at −20°C for later processing.

Meteorological/environmental parameters (temperature, absolute wind speed and wind direction, global radiation, longwave radiation, air pressure, and humidity) were recorded with the DAVIS-Ship FS on board. Measurements were recorded in 5-min intervals and arithmetically averaged for the period of 1 h.

### DNA extraction

Following the procedure of Sapp et al. (2006), a chemical cell lysis with an additional freeze–thaw step (Maron et al. 2005) and a subsequent phenol–chloroform extraction was performed. The total volume of 20 *μ*L of DNA extract per sample was obtained. Concentration and pureness of the extracted DNA were then determined photometrically using the microplate reader Tecan Infinite© 200 from NanoQuant photometry (Tecan, Männedorf, Switzerland). All DNA was stored at −20°C for later use.

### 16S rRNA gene amplicon pyrosequencing

All 36 samples were applied to pyrosequencing of the 16S rRNA gene. The amplicon library was constructed with primers of the structure 5′-(Roche's adaptor for long reads [Lib-L])–(template-specific sequence)-3′. As template-specific sequences we used forward primer GM3 (5′-AGAGTTTGATCMTGGC-3′) and reverse primer 907 (5′-CCGTCAATTCMTTTGAGTTT-3′) (Krause et al. 2012). Due to the small yield of PCR product with the pyrosequencing tagged primer, probably due to low DNA content in the samples, a reamplification PCR for the prokaryotic 16S rRNA gene was conducted. In a first PCR reaction, the eubacterial forward primer GM3 (5′-AGAGTTTGATCMTGGC-3′) and the reverse primer 907 (5′-CCGTCAATTCMTTTGAGTTT-3′) (Muyzer et al. 1995) were applied with each 5-ng DNA as template. Subsequently, 1 *μ*L of the reaction was used for a PCR with pyrosequencing tagged primers. PCR reactions were run with 2.5 *μ*L taq buffer (10×), 5 *μ*L TaqMaster PCR Enhancer® (5×), 0.7 *μ*L of each primer (20 mmol/L), 0.75 *μ*L dNTPs (2.5 mmol/L each), and 3 U of taq DNA polymerase (5 Prime, Hamburg, Germany), and were adjusted with ultrapure water to a reaction volume of 25 *μ*L. The conditions for both PCR reactions (with and without pyrosequencing tagged primers) were the following: 94°C for 10 min, followed by 30 cycles with 94°C for 1 min, 44°C for 1:30 min, and 68°C for 2 min; final elongation at 68°C for 5 min. PCR products were obtained for 31 of the 36 collected samples. PCR products were then verified by gel electrophoreses and by the absence of amplifications in DNA-free controls. PCR products were purified with the peqGOLD Gel Extraction Kit (Peqlab, Erlangen, Germany). Sequencing was done on a Roche 454 GS-FLX Titanium platform (LGC Genomics, Berlin, Germany).

All sequence reads were processed on the NGS analysis pipeline of the SILVA rRNA gene database project (SILVAngs; Quast et al. 2013). Each read was aligned against the SILVA SSU rRNA SEED and quality controlled, using the SILVA Incremental Aligner (SINA) (Pruesse et al. 2012); (Quast et al. 2013): reads shorter than 50 aligned nucleotides and reads with more than 2% of ambiguities, or 2% of homopolymers, were excluded from further processing. Suspected contaminations and artefacts as well as reads with a low alignment quality (50 alignment identity, 40 alignment score reported by SINA) were also identified and excluded from downstream analysis.

After these initial steps of quality control, identical reads were identified in a dereplication step. Unique reads were clustered on a per sample basis (operational taxonomic unit/OTU), and the reference read of each OTU was classified. Dereplication and clustering were conducted using cd-hit-est (version 3.1.2; http://www.bioinformatics.org/cd-hit; Li and Godzik 2006) with identity criteria of 1.00 and 0.98. For classification, local nucleotide BLAST was compared to the nonredundant version of the SILVA SSU Ref dataset (release 111; http://www.arb-silva.de) using blastn (version 2.2.22+; http://blast.ncbi.nlm.nih.gov/Blast.cgi; Camacho et al. 2009). The obtained classification of the OTU reference reads were each mapped onto all reads that had been assigned to the respective OTU. Reads without any BLAST hits and reads with only weak BLAST hits function “(% sequence identity + % alignment coverage)/2” not exceeding a value of 93) remained unclassified. These reads were assigned to the meta group “No Relative” in the SILVAngs fingerprint and Krona charts (Ondov et al. 2011). The remaining classified sequences formed an OTU table and singletons were excluded from the analyses. Although nonbacterial sequences, chloroplast and mitochondrial sequences were recorded; they were not further analyzed. Only phyla, classes, and families with an occurrence of 1% or higher within the respective groups were considered for the heatmap construction. All sequence data were deposited at the NCBI Sequence read archive (accession number: SRP043406).

### Air mass backward trajectories

In order to determine the origin of a given air parcel, backward trajectories of all sampled air masses were created using the HYSPLIT model (HYbrid Single-Particle Lagrangian Integrated Trajectory model; Draxler and Rolph 2013). Five-day backward trajectories (commonly used in bioaerosol studies) were calculated for air parcels arriving at three different arriving heights a.s.l., each representing a different air layer: 10 m (sampling height, ground influence), 500 m (the upper part within the boundary layer), and 1500 m (above boundary layer) a.s.l.. This was done for each sampling period, detecting possible mixing events between different air layers. The midway of each transect was chosen as the starting point and the start time for the corresponding trajectory.

### Statistical analysis

All statistical analyses were performed with the computer software packages PRIMER 6 (with PERMANOVA+ ad-on; PRIMER-E Ltd, Ivybridge, United Kingdom) and GraphPad Prism 5.04 (GraphPad Software, Inc., La Jolla, USA). Alpha diversity for each sample was expressed by Simpson's diversity index with 1 − *λ*′ = 1 − ∑(*n*_i_ × (*n*_i_ − 1)/(*N* × (*N* − 1)) as it provides good estimate of diversity at relatively small sample sizes without assumptions about underlying distribution of species abundance (Magurran 2004). Obtained alpha diversity values were compared with regard to different environmental factors (see Table1 for abbreviation), being the following: “sampling location” (factor levels: “NS”, “SK”, “KT”, “BS,” and “KC”), “wind direction” (factor levels: “N,” “E,” “SE,” “S,” “SW,” “W,” “NW”), “backward trajectory influence” (BWT Influence factor levels: marine, more marine (75%), mixed, more continental (75%), continental), “backward trajectory” (BWT factor levels: no crossing, no height; crossing, no height; crossing, height; no crossing, height), “cardinal direction” (factor levels: “N,” “E,” “S,” “W”), “height” (factor levels: low; 500; 1000; 1500; 2000 m a.s.l), and “rain” (factor levels: no rain [N]; rain during sampling [0]; rain 6 h before sampling [6]; rain 60 h before sampling [60]) (see Table S1). Due to the different amount of samples within each factor level, the data did not fulfill the requirements of an ANOVA (Kolmogorov–Smirnov test, Bartlett's test). Therefore, the nonparametric Kruskal–Wallis test was performed followed by Dunn's post hoc procedure.

**Table 1:** Abbreviations

Group	Abbreviation
North	N
East	E
Southeast	SE
South	S
Southwest	SW
West	W
Northwest	NW
North Sea	NS
Skagerrak	SK
Kattegat	KT
Baltic Sea	BS
Kiel Canal	KC

Bray–Curtis similarities were calculated for the square root transformed OTU abundance data (equalization of different sample size) and visualized by principal coordinate analysis (PCO, Krause et al. 2012). Beta diversity was then analyzed with a permutational multivariate ANOVA (PERMANOVA; Anderson 2001) with the same factors as for alpha diversity (see above).

A distance-based linear model (DistLM) with distance-based redundancy analysis (dbRDA) visualization was performed on the Bray–Curtis similarities of the OTU abundances with Euclidean distances of the environmental parameters (predictor valuables). This allowed for modeling the relationship of the OTU abundance data and environmental parameters in order to test for possible influences of the environmental parameters. Environmental parameters (global radiation; longwave radiation; absolute wind direction; absolute wind speed; air pressure; air temperature; humidity) were log(*x* + 1) transformed, normalized, and subjected to a principal component analysis (PCA) to visualize patterns of the relationship of the different environmental parameters.

## Results

### Weather conditions and backward trajectories

Weather conditions during the sampling period were mild (13–21°C), humid (69–96%), and wind speed varied from 1 to 15 m/sec (Table S2).

**Figure 2:**
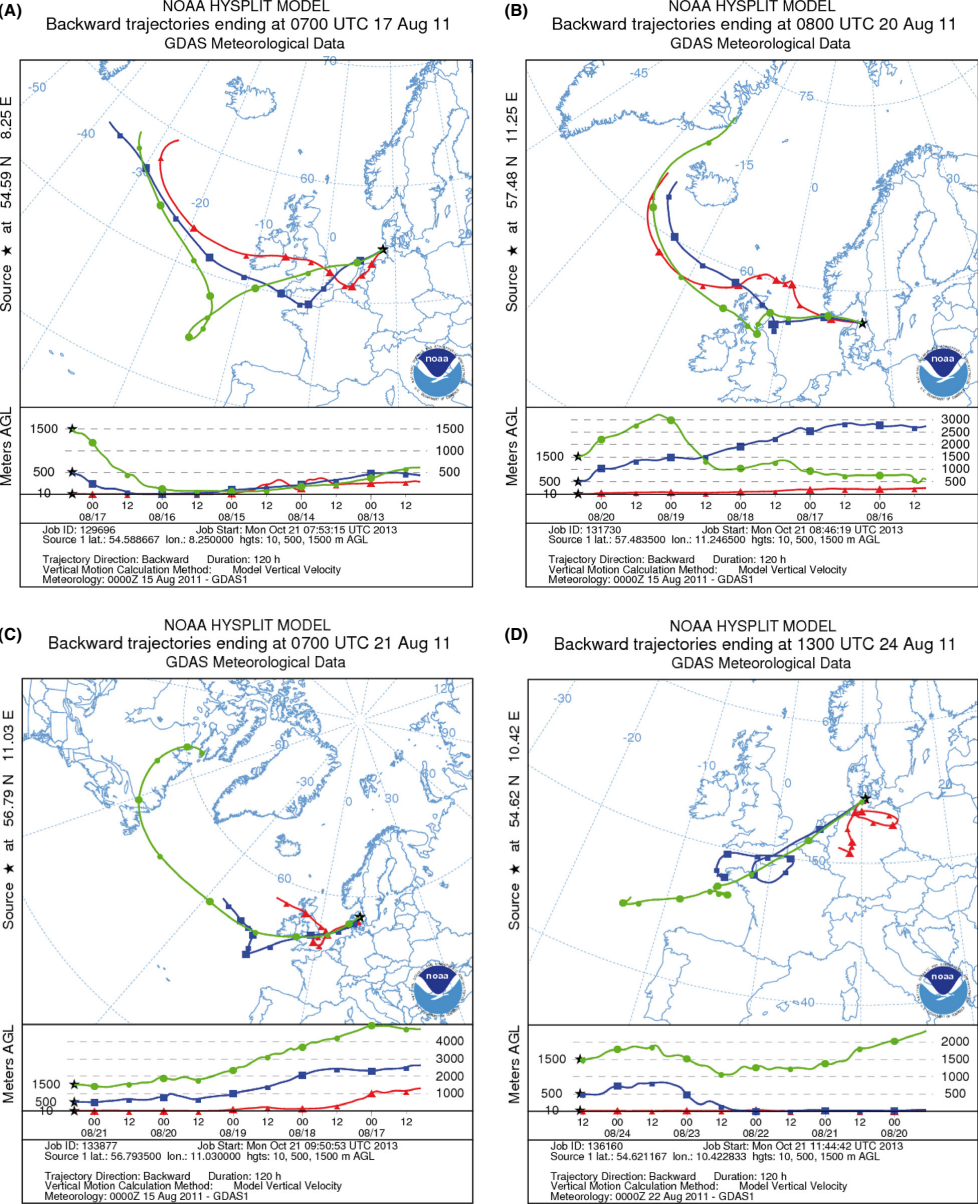
Backward trajectories calculated with the NOAA Hysplit Model (Draxler and Rolph 2013) with three different transects with different arriving heights: red = 10 m; blue = 500 m; green = 1500 m. Sampling time and place are indicated by the black asterix (A = Sample 1; B = Sample 14; C = Sample 17; D = Sample 31).

Five-day backward trajectories were calculated for each sample with three transects which were characterized by different arriving heights (10 m, 500 m, 1500 m). In all sampled air parcels, the origins of the backward trajectories were highly variable, but western and southern directions clearly dominated. Based on these backward trajectories, there were no direct eastern winds. Samples with strong marine influence (e.g., sample 14; Fig.2B) were always characterized by air masses with projected trajectories which were close to the sea surface. However, also samples with air masses of mixed origin (50% continental influence, 50% marine influence) (e.g., sample 1) were observed (Fig.2A). For the majority of samples, the origin of air parcels samples was consistent with the general wind direction recorded by the DSHIP system. In few samples, the origin differed from the recorded wind direction (e.g., 17 and 31; Fig.2C and D). For example, the eastern wind direction during the sampling at location 31 was in clear contrast to the backward trajectories which showed a strong southern/continental influence. The 5-day back-calculation of air parcel movements showed a consistent movement close to the ground over the German mainland. The air parcel of sample 17 started in the western direction over the North Atlantic and crossed Scotland, the North Sea and Denmark before the sampling in the Kattegat from a southern direction.

### Bacterial diversity

A total number of 293,011 reads was obtained after quality check via SILVAngs databank. Chloroplast sequences (125,993 = 43%) and mitochondrial sequences (49,607 = 17%) made up 60% of all sequences. The proportion of chloroplast and mitochondrial sequences varied between the samples (Table S8). Chloroplast sequence abundances ranged from 2.2% to 88.5% and mitochondrial sequences from 0.1% to 69.2%. Chloroplast and mitochondrial sequences as well as singletons (*n* = 1) were omitted from the analysis of the bacterial reads.

The remaining 117,232 bacterial sequences were highly variable among the samples and ranged from 8 to 11,537 sequences per sample (Table S8). Sample 27 (*n* = 8) was excluded from analysis due the small number of sequences. The bacterial sequences were classified in 753 OTUs. The rarefaction curves did not reach an asymptote for all samples, suggesting that the full extent of airborne bacterial diversity could not be recovered for all samples.

The calculated diversity values (Simpson's diversity index) showed no significant differences for most of the considered factors (BWT: *H*_K-W_ = 7.663, *P* = 0.1048; BWT influence: *H*_K-W_ = 6.621, *P* = 0.0850; cardinal direction: *H*_K-W_ = 3.798, *P* = 0.2841; wind direction: *H*_K-W_ = 8.779, *P* = 0.1864; height: *H*_K-W_ = 2.650, *P* = 0.6180; rain: *H*_K-W_ = 4.062, *P* = 0.2548). However, the factor “sampling location” had a significant effect on the bacterial diversity (*H*_K-W_ = 12.45, *P* = 0.0143). This was particularly due to the differences between “KC” and “KT” (*P* < 0.05).

The PCO plot with 23.8% explained variation for the *x*-axis and 11.1% for the *y*-axis (34.9% in total), showed no clear patterns or grouping (Fig. S1). However, the PERMANOVA of beta diversity revealed significant effects for the factors “sampling location” (*P* = 0.048), “cardinal direction” (*P* = 0.040), and “wind direction” (*P* = 0.001; Table2). Pairwise comparisons for “sampling location” showed significant differences between the samples from the “BS” and the “NS” (*P* = 0.049) as well as the “SK” (*P* = 0.0437; Table S3). In detail, the significant effect for “cardinal direction” was caused by differences between “east” and “west” (*P* = 0.043) as well as between “E” and “N” (*P* = 0.0437; Table S4). Comparison of the wind directions revealed significant differences of “SW” in direct comparison to “W” (*P* = 0.010) and to NW (*P* = 0.002) as well as between “W” and “E” (*P* = 0.031; Table S5). The factor “rain” also had a significant effect on the beta diversity (*P* = 0.035; Table2) although this could not be supported by pairwise comparisons (Table S6). The factors “BWT,” “BWT influence,” and “height” had no significant effect on the bacterial diversity (BWT: *P* = 0.507; BWT Influence: *P* = 0.123; Height: *P* = 0.274; Table2).

**Table 2:** PERMANOVA main test of bacterial community composition based on Bray–Curtis dissimilarities of OTUs (16S rRNA gene amplicon sequencing)

Group	df	SS	Pseudo-*F*	*P* (perm)^1^	Sq. root
Sampling location	4	9998.4	1.3389	**0.048**	11.103
BWT	4	8921.6	1.2114	0.123	8.1096
BWT Influence	3	5432.3	0.95196	0.507	−4.8722
Cardinal direction	4	7948.0	1.4646	**0.04**	11.648
Wind direction	6	16,674	1.6626	**0.001**	16.148
Height	4	826,208	1.1068	0.274	7.4099
Rain	3	7928.1	1.4603	**0.035**	11.214

Displayed are tests for the factors “BWT,” “BWT Influence,” “Cardinal direction,” “Wind direction,” “Height,” and “Rain” and the partitioning of multivariate variation. *p*-values were obtained using type III sums of squares and 999 permutations under the full model. df, degrees of freedom; SS, sums of squares; Sq. root, square root of the component of variation attributable to that factor in the model, in units of Bray–Curtis dissimilarities; BWT, backward trajectory influence.

1 Significant results (*P* (perm)* *< 0.05) are highlighted in bold.

PCA of environmental parameters displayed an even distribution of the individual sampling sites (Fig. S2). However, there was a clear separation when the geographical group factors were taken into account. Within the data, 32.1% of the variation was accounted to PC1 and 25.6% was accounted to PC2. Therefore, 57.7% of the variation could be explained by these two components (Table S7).

**Figure 3:**
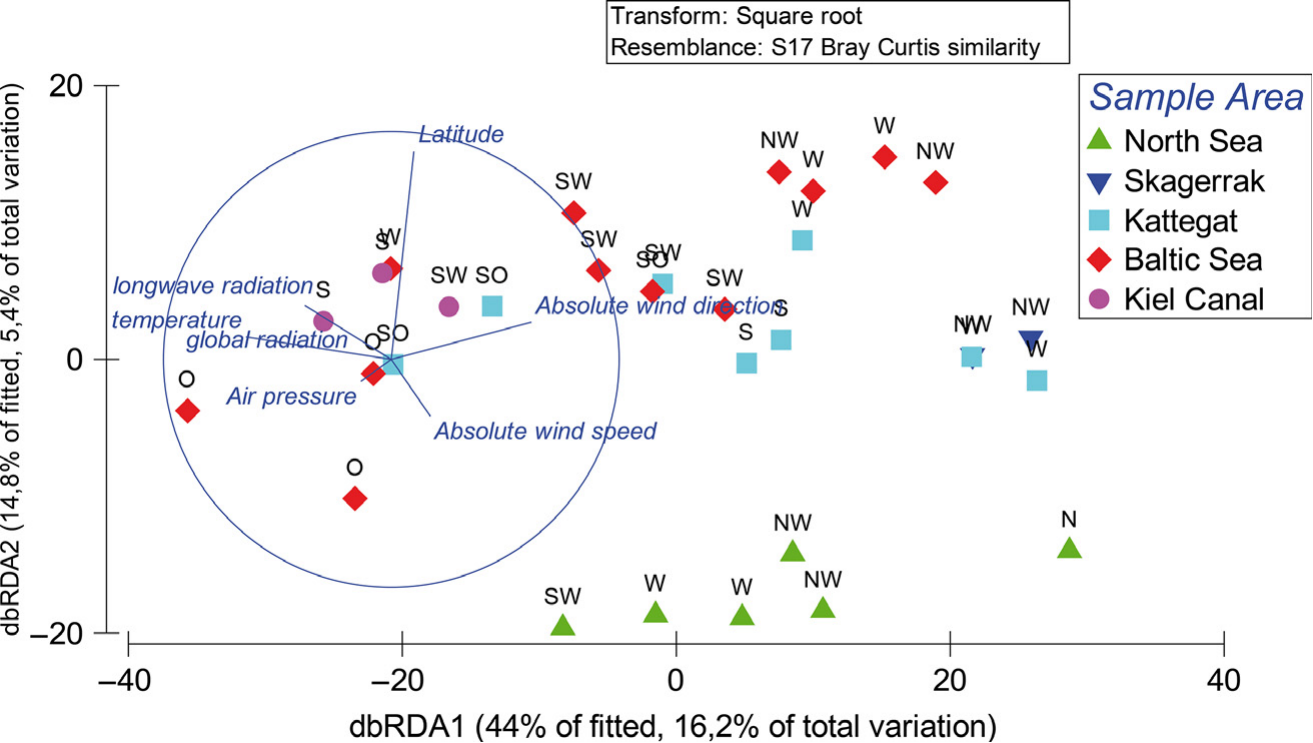
dbRDA ordination relating environmental variables with the OTU composition of samples taken. The “% of fitted” indicates the variability in the original data explained by the fitted model and “% of total variation” indicates the variation in the fitted matrix.

DistLM analysis revealed significant effects of the factors “longwave radiation” (*P* = 0.023), “absolute wind direction” (*P* = 0.002), “absolute wind speed” (*P* = 0.020), “air temperature” (*P* = 0.020) and “longitude” (*P* = 0.017; Table3). The sequential test showed that the influence of temperature (*P* = 0.001), longwave radiation (*P* = 0.02), latitude (*P* = 0.033), and wind direction (*P* = 0.024) contributed significantly to the explained variation (Table4). The first axis of the dbRDA explained 44% of fitted and 16.2% of total variance, whereas the second axis explained 14.8% of fitted and 5.6% of total variance (Fig.3). The separation along the *x*-axis was due to the factors “wind direction” and “temperature,” which were negatively correlated, and due to latitude along the second axis.

**Table 3:** Distance-based linear models (DISTLM) calculation for the influence of environmental variables on OTU composition

Variable	Pseudo-*F*	*P*^1^	Prop.
Global radiation	1.0319	0.359	3.44E-02
Longwave radiation	2.0764	**0.023**	6.68E-02
Absolute wind direction	3.2902	**0.002**	0.1019
Absolute wind speed	1.9765	**0.020**	6.38E-02
Air pressure	1.4957	0.099	4.90E-02
Air temperature	4.0158	**0.001**	0.12163
Humidity	1.2687	0.168	4.19E-02
Longitude	2.1305	**0.017**	6.84E-02
Latitude	1.4607	0.093	4.80E-02

1 Significant results (*P* (perm)* *< 0.05) are highlighted in bold.

**Table 4:** Sequential test of distance-based linear models (DISTLM) calculation

Variable	Pseudo-*F*	*P*^1^	Proportion of variance	Cumulation
+Air temperature	4.0158	**0.001**	0.12163	0.12163
+Longwave radiation	1.7393	**0.020**	5.14E-02	0.173
+Latitude	1.5829	**0.033**	4.58E-02	0.2188
+Absolute wind direction	1.7254	**0.024**	4.86E-02	0.26742
+Air pressure	1.3306	0.137	3.70E-02	0.30444
+Absolute wind speed	1.2629	0.167	3.48E-02	0.33921
+Global radiation	1.0213	0.431	2.81E-02	0.3673

Tests for relationship between the OTU composition for different samples with environmental parameters. Amount explained by each variable added to model is conditional on variables already in the model.

1 Significant results (*P* (perm)* *< 0.05) are highlighted in bold.

### Pyrosequencing data analysis

The 37 most abundant OTUs, each present with more than 500 sequences, made up more than 76% of all sequences (Table S9). The remaining 716 OTUs comprised 24% of the sequences. The most abundant genus *Sphingomonas* was present with 17% of all bacterial reads.

The statistical analyses of the bacterial community structure (DistLM and PERMANOVA) revealed a significant influence of the factor “wind direction” and “sampling location” (Table4, Fig.3). To further analyze these influences and to identify the responsible bacteria, all samples were grouped by the factors “wind direction” and “sampling location” and compared with each other.

The number of samples comprised by “wind direction” groups ranged from 1 to 8 and the sequences represented by each wind direction group ranged from 756 to 27,955. The number of OTUs ranged from 96 to 573 per wind direction (Table5).

**Table 5:** Number of samples, sequences, and OTUs belonging to the different wind direction groups

Group	Samples	Sequences	OTUs	Sample ID
North	1	756	96	H08
East	3	16,292	388	H26, H28,H 32
Southeast	3	13,182	388	H19, H20, H25
South	4	20,364	444	H17, H18, H35, H36
Southwest	6	27,955	573	H01, H16, H29, H30, H33, H34
West	8	21,486	447	H02, H03, H13, H14, H15, H21, H24, H31
Northwest	6	17,189	401	H04, H06, H11, H12, H22, H23

Samples per “sampling location” ranged from 2 to 12 samples, with 4945 to 46,079 sequences and 221 to 578 OTUs per location (Table6).

**Table 6:** Number of samples, sequences, and OTUs belonging to the different wind direction groups

Group	Samples	Sequences	OTUs	Sample ID
North Sea	6	25,140	533	H01, H02, H03, H04, H06, H08
Skagerrak	2	4945	221	H11, H12
Kattegat	8	31,786	511	H13, H14, H15, H16, H17, H18, H19, H20
Baltic Sea	12	46,079	578	H21, H22, H23, H24, H25, H26,H 28, H29, H30, H31, H32, H33
Kiel Canal	3	9274	452	H34, H35, H36

The phylogenetic analysis of bacterial communities was restricted to phyla, classes, and families with an OTU occurrence of 1% or higher. In the following, the relative abundance of each group represents its proportion within the respective taxonomic groups.

**Figure 4:**
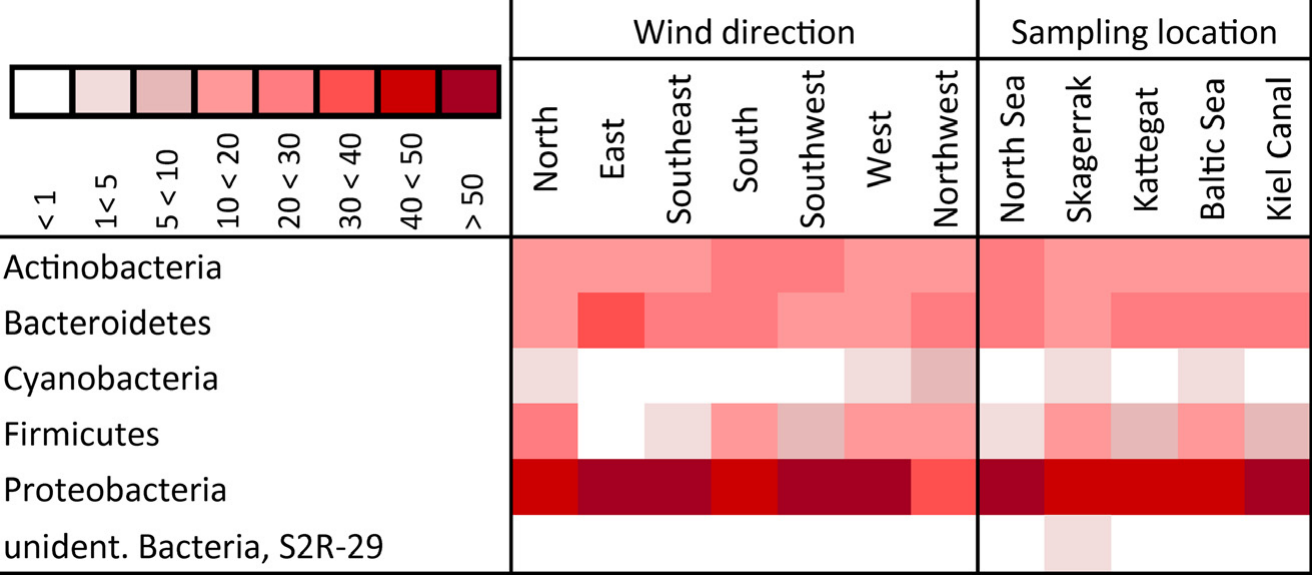
Taxonomic classification of bacterial reads grouped in wind direction and sampling location on phyla level using SILVA classifier based on 98% similarity omitting singletons (*n* = 1) and rare reads (<1%). The amount of percentage proportion contribution of each phyla per group is indicated by color of cell; darker color represent higher contribution.

Four important phyla were detected in the aerosol samples: Actinobacteria (16.25%), Bacteroidetes (22.87%), Firmicutes (8.33%), and Proteobacteria (49.28%) (Fig.4). In combination with the Cyanobacteria (1.43%), the four main phyla represented 98.17% of all bacterial reads. The remaining bacterial sequences were affiliated with the classes Acidobacteria, Aquificae, Armatimonadetes, Chloroflexi, Deinococcus-Thermus, Fusobacteria, Gemmatimonadetes, Lentisphaerae, Nitrospirea, Planctomycetes, Tenericutes, and Verrucomicrobia.

Proteobacteria were the dominant phylum in the dataset with an even distribution among the “wind direction” and “sampling location” with ca. 50% of all sequences (except for the wind directions: “N” 45%, “S” 43%, “NW” 36%, and the sampling location “SK”: 45%).

Cyanobacteria were restricted to the wind directions “N” (1.5%), “W” (1.6%), and “NW” (6.7%) and to “SK” and “BS” locations (2–3%).

Most abundant Proteobacteria were Alphaproteobacteria with *Sphingomonas*, as mentioned above, accounting for 17% of all bacterial sequences, *Anderseniella* (3.87%), and *Rhizobium* (3.36%). Within the Betaproteobacteria, *Massilia* was most abundant with 2.83% of all sequences, whereas *Pseudoalteromonas* (2.79%), *Psychrobacter* (2.79%), and *Pseudomonas* (1.56%) were the most frequent Gammaproteobacteria.

Most abundant Bacteroidetes were *Prevotella* (2.38%; Bacteroidia), *Hymenobacter* (4.68%; Cytophagia), as well as the two Flavobacteria *Brumimicrobium* (1.76%) and *Chryseobacterium* (1.77%).

The most frequent Firmicutes sequence was the Bacilli *Staphylococcus* (3.72%) and the most frequent Actinobacteria were unidentified Microbacteriaceae (2.39%), *Curtobacterium* (2.40%), *Arthrobacter* (2.22%), and *Propionibacterium* (2.40%).

Comparing the distribution of grouped sequences on phylum level, an influence of the factor “wind direction” was visible. Proteobacteria, Bacteriodetes, and Actinobacteria were more or less evenly distributed among “wind directions” and “sampling locations.” In contrast, Firmicutes sequences were nearly absent from “E” winds but were detected at all sampling locations (“NS,” “KC” and “KT” 4–8%, “BS” 11% and “SK” 19.5%).

#### Proteobacteria

**Figure 5:**
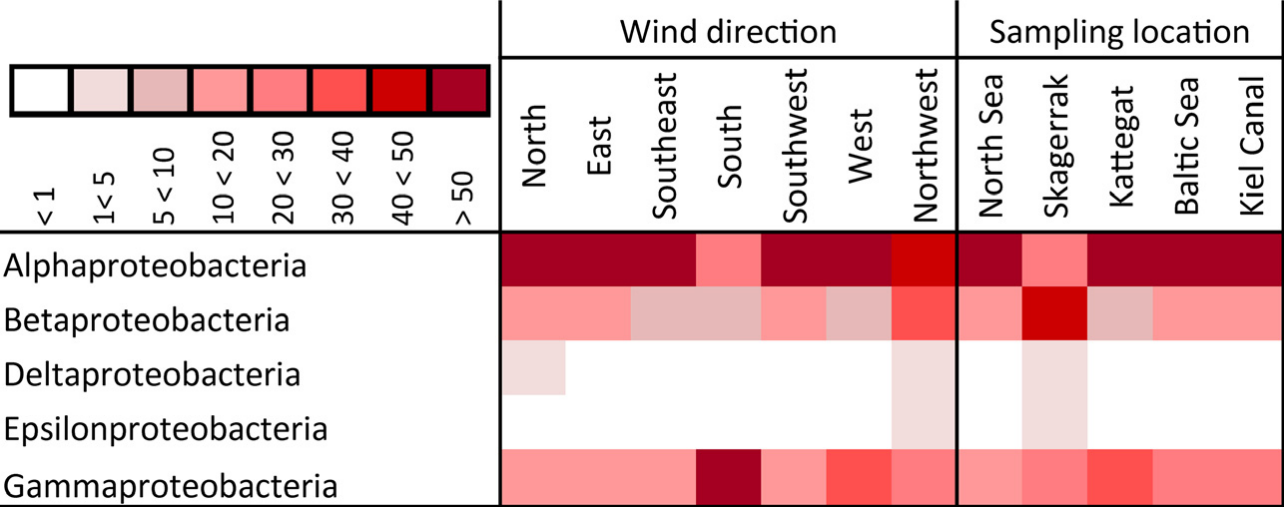
Taxonomic classification of Proteobacteria reads grouped in wind direction and sampling location on class level using SILVA classifier based on 98% similarity omitting singletons (*n* = 1) and rare reads (<1%). The amount of percentage proportion contribution of each class per group is indicated by color of cell; darker color represents higher contribution.

Within the Proteobacteria (Fig.5), the three main classes Alpha-, Beta-, and Gammaproteobacteria were found with Alphaproteobacteria being the most abundant class.

The comparison of the two groupings revealed an influence of the factor “wind direction” on the distribution of Gammaproteobacteria as well as an influence of the “sampling location” on the Betaproteobacteria. Gammaproteobacteria sequences were evenly distributed among the sampling locations but showed a dominance in “S” wind samples (61% of Proteobacteria sequences in this wind direction group), whereas Betaproteobacteria showed an even distribution among the wind directions with a dominance in “SK” location samples (45% of all Proteobacteria sequences at this location group).

**Figure 6:**
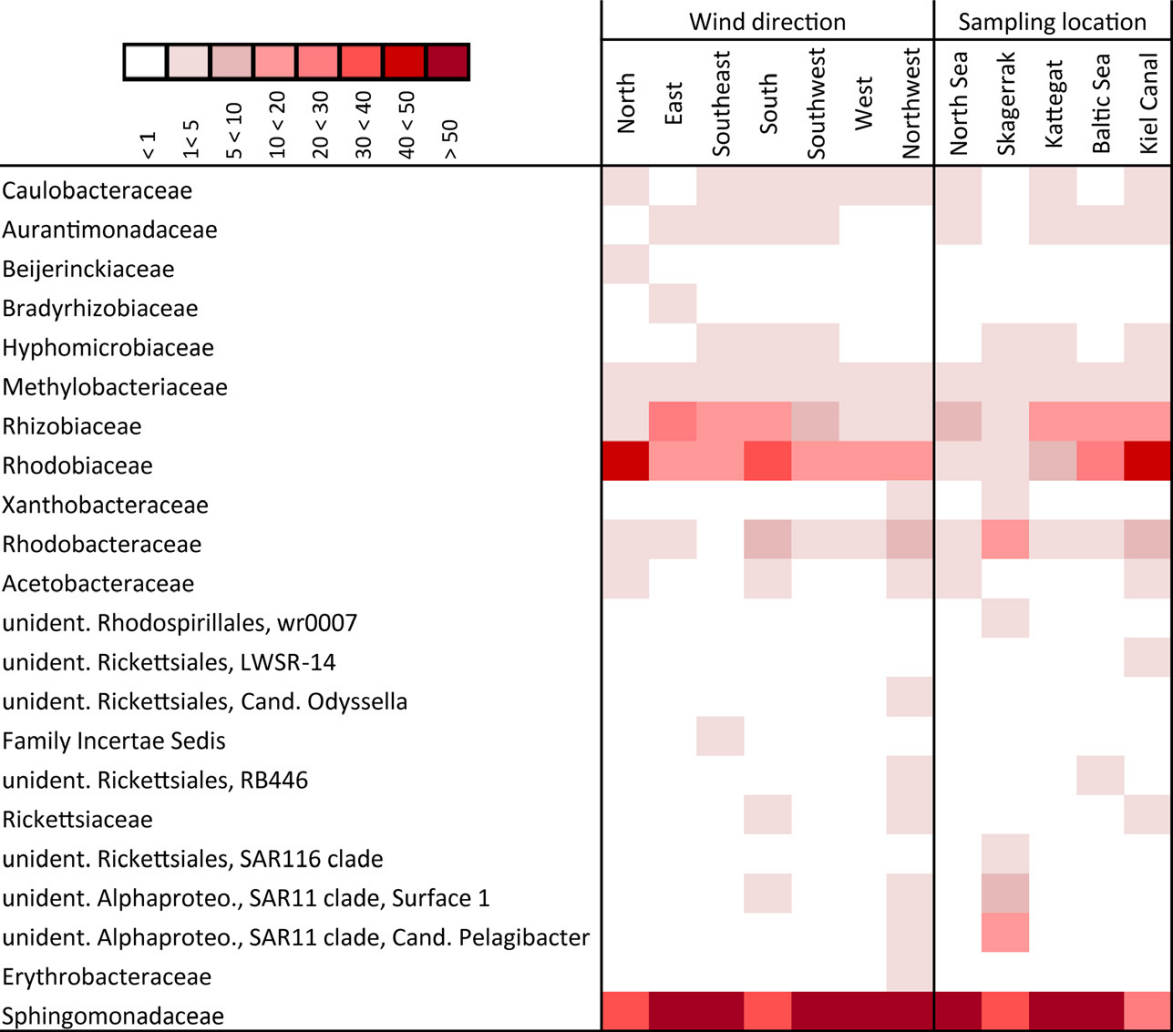
Taxonomic classification of Alphaproteobacteria family reads grouped in wind direction and sampling location on class level using SILVA classifier based on 98% similarity omitting singletons (*n* = 1) and rare reads (<1%). The amount of percentage proportion contribution of each family per group is indicated by color of cell; darker color represents higher contribution.

Alphaproteobacteria were represented by 22 families (Fig.6). The highest diversity was observed in the “NW” group with 14 detected families, followed by “S,” “SK,” and “KC” with 11 families. The most dominant family was Sphingomonadaceae represented by the genus *Sphingomonas*, also being the most abundant OTU in the entire dataset. Only in the “N,” “S,” and “KC” group, the family Rhodobiaceae dominated with the genus *Anderseniella*. Although Rhodobiaceae were also present in other groups, they occurred in much lower numbers. An influence of the sampling location on the distribution of Rhodobiaceae was observed. In “NS” and “SK” samples they made up only 2% and 4% of all sequences but gradually increased from the “KT” (7.4%) to the “BS” (21.3%) and the “KC” (40.4%) while they were more evenly distributed among the “wind direction.”

Betaproteobacteria comprised nine families (Fig. S3). The dominant Oxalobacteraceae (genera *Massilia* and *Oxalobacter*) represented more than 50% of all sequences in all groups, except for the “NW” and “N” group but were still the most abundant family. Comamonadaceae (genera *Variovorax* and *Acidovorax*) were evenly distributed to all wind directions and locations (13–29%) but were found in low numbers in “SK” samples (6.5%). However, no influence was found on the family level of Betaproteobacteria for the factors “wind direction” and “sampling location”.

The class Gammaproteobacteria comprised 20 families with no dominant family/genus for all samples (Fig. S4). Moraxellaceaea (*Psychrobacter*,*Acinetobacter*,*Enhydrobacter*) were present in all sample groups, dominating in “W” (63%) and “NW” (35%) and also in the “NS” and “BS” samples (42–48%). Pseudoalteromonadaceae (*Pseudoalteromonas*) were found in all samples in comparably low numbers (3–5%). However, clear dominances in “S” (50%), “KT” and “KC” (42–45%) were detected. Enterobacteriaceae (*Pantoea*, Escherichia-Shigella, *Citrobacter*) were found in all groups with higher abundances in “N” and “E” (25–30%). In the “NS,” “SK,” and “KT” (6–7%), however, abundances were slightly lower than in the “BS” and the “KC” (12.5–14.7%). Pseudomonadaceae (*Pseudomonas*) were present in all groups dominating in “E” and “SE” (42–44%) as well as in the “SK” (36.9%). Abundances in “S,” “W,” and “KT” (5.3–7.9%) were comparably low.

#### Actinobacteria

The phylum Actinobacteria was strongly dominated by the class Actinobacteria. Furthermore, in the groups “W,” “NW,” “N,” “E,” “SW,” the class Acidimicrobia was detected in low numbers (3–10%). In addition, Acidimicrobia was detected at all locations (1.5–2.5%) and in the two “SK” samples Acidimicrobia made up 30.8% of the Actinobacteria sequences. Therefore, an influence of sampling location was detected on class level for the phylum Actinobacteria.

**Figure 7:**
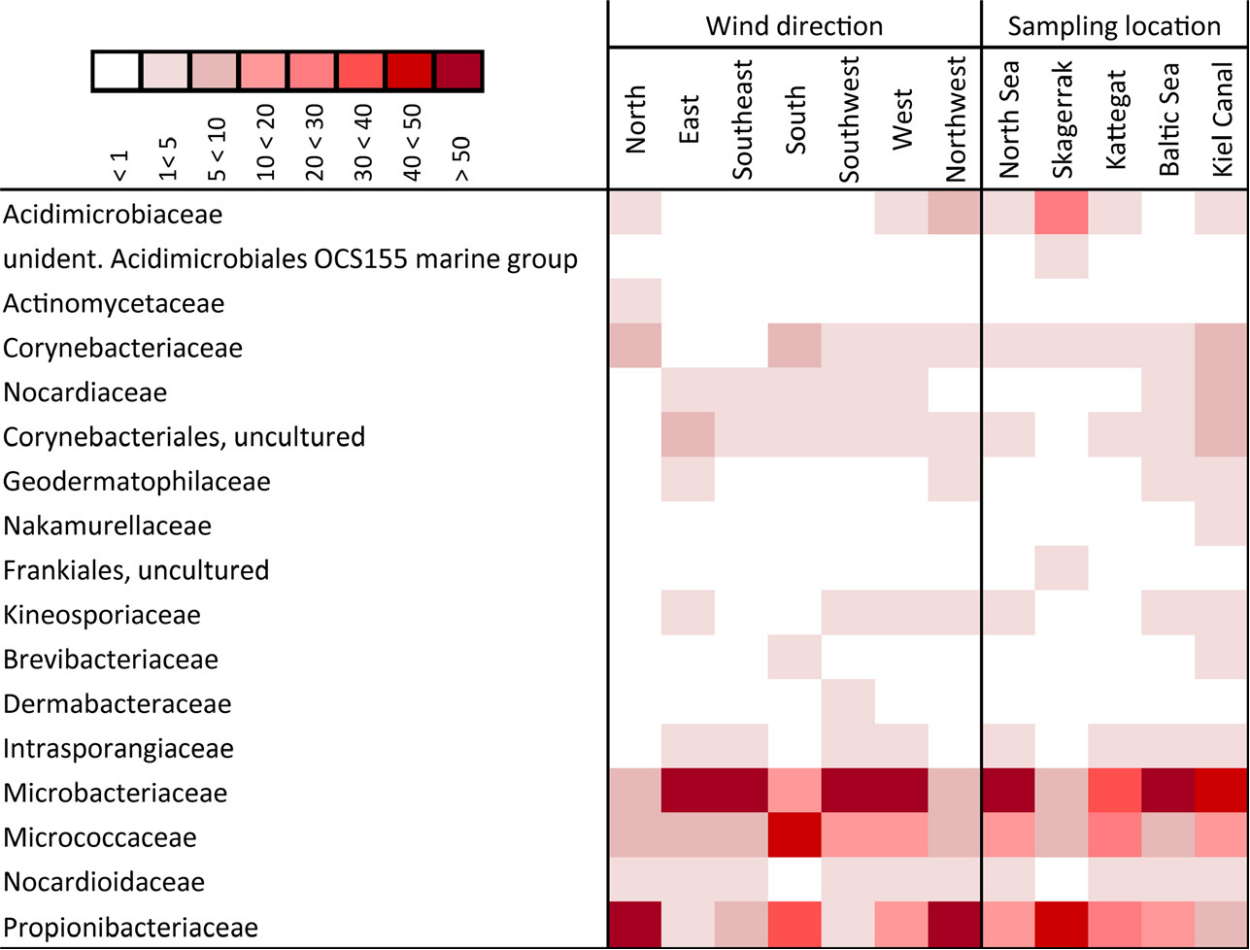
Taxonomic classification of Actinobacteria reads grouped in wind direction and sampling location on family level using SILVA classifier based on 98% similarity omitting singletons (*n* = 1) and rare reads (<1%). The amount of percentage proportion contribution of each family per group is indicated by color of cell; darker color represents higher contribution.

The Actinobacteria comprised 18 families (Fig.7) with Propionibacteriaceae (*Propionibacterium*,*Friedmaniella*), Micrococcaceae (*Arthrobacter*), and Microbacteriaceae (*Curtobacterium*, unident. Microbacteriaceae, *Agreia*,*Clavibacter*;*Microbacterium*,*Plantibacter*) with high abundances in all considered groups. Coryneobacteraceae were not present in “E” and “SE” direction but were found at all locations.

#### Firmicutes

Firmicutes were dominated by the class Bacilli and Clostridia (Fig. S5). Within the class Clostridia, 13 families were mostly even distributed (Fig. S6). Clostridiaceae (*Clostridium*) was the dominant family in every group except for one sample from North, where the family XI Incertae Sedis was dominant (60%). For the other wind directions, abundances were lower (1.5–4.1%) which was similar in the location groups with slightly higher abundances in samples from the “KC” and the “NS” (8–15%). Lachnospiraceae and Rumicocaceae were constantly detected in every group.

The detected 14 families of the class Bacilli were strongly dominated by Staphylococcaceae (genus *Staphylococcus*; 53–91%, except for “E”: 33%; Fig. S7). The two families Bacillaceae (*Bacillus*,*Aeribacillus*) and Streptococcaceae (*Streptococcus*) were also present in all groups. Bacillaceae (5–11%, except for “E”: 30%) and Streptococcaceae (12–18%, except for “N” and “SW”: 2% and “NS,” “SK,” and “KC” 5.7–8.4%) showed an even distribution among the samples.

#### Bacteroidetes

The phylum Bacteroidetes comprised four main classes which were present in all groups (Figs. S8, S9). The class Bacteroidia was represented by the three families Bacteriodaceae, Porphyromonadaceae, and Prevotellaceae. Prevotellaceae (genus *Prevotella*) was the most abundant Bacteroidia and was found to be evenly distributed among all “wind direction” and “sampling location” groups, clearly dominating in the “N” (67%) group. Two families were found in the class Cytophagia: the Cyclobacteriaceae and the Cytophagaceae. Cyclobacteriaceae (*Algoriphagus*) were only found in “W” samples and the “KT” in low numbers, whereas Cytophagaceae (*Hymenobacter*,*Dyadobacter*,*Spirosoma*) were present in all groups. Flavobacteria dominated in the “S” directions (61%) and the “KT” location (45.9%) and were evenly distributed between the other locations and wind directions (9.9–24.3%). The class Flavobacteria comprised two families: Flavobacteriaceae were present in all wind direction and location groups in equal abundances, whereas Cryomorphaceae were restricted to “S” (41%) and “NW” (<2%) wind directions as well as the “SK” (1.4%), “KT” (26.3%) and the “KC” (3.7%). The class Sphingobacteriia was comprised of two families which were found in all groups. The most abundant Sphingobacteriaceae (*Pedobacter*), and Chitinophagaceae were found in low abundances in all groups (location and Wind direction) except for “NW” wind directions.

## Discussion

### Bacterial diversity

The pyrosequencing reads contained a high amount of mitochondrial and chloroplast sequences. Chloroplast sequences are a common element of bioaerosol samples, particularly in the summer months, and have been reported from numerous studies (e.g., Maron et al. 2005; Brodie et al. 2007; Fahlgren et al. 2010, 2011; Franzetti et al. 2011; Jeon et al. 2011; Zweifel et al. 2012; Bertolini et al. 2013), regardless of the applied molecular technique. The occurrence of mitochondrial sequences may be explained by aerosolized microalgae and plant debris which most likely also caused the high amount of chloroplast sequences. There is a distinct lack of information about mitochondrial sequences in comparable studies, which could be due to different sampling techniques. Sampling methods in other investigations ranged from direct vacuum filtration on filters (Bowers et al. 2009, 2011a; Fahlgren et al. 2010), low volume gravimetric samplers (Franzetti et al. 2011) and commercial vacuum cleaners (Zweifel et al. 2012), to high volume air samplers (Jeon et al. 2011), and wetted-wall cyclone samplers (Maron et al. 2005). Another explanation could be that mitochondrial sequences were simply disregarded in other studies.

The pyrosequencing revealed a high bacterial diversity in marine bioaerosols, which could not be accessed in total (deduction from the rarefaction curves). So even with our high-throughput approach to gain as many reads as possible with 3781 sequences on average per sample we could not survey the full extent of airborne bacterial community diversity with our data set. Overall, four important phyla were detected: Proteobacteria, Bacteroidetes, Firmicutes, and Actinobacteria. The gram-negative bacteria Proteobacteria and Bacteroidetes made up 72.15% of all bacterial reads, whereas the gram-positive bacteria Firmicutes and Actinobacteria were less frequent with only 24.58%. This conforms to the findings of other studies, which used molecular approaches (see Després et al. 2012 and references therein).

Furthermore, 37 OTUs made up more than 75% of all sequences, implying the existence of a dominant core community of bacteria with a long tail of rare OTUs. A similar assemblage of bacteria was also reported by Fahlgren et al. (2010). According to Gandolfi et al. (2013), there may be a bacterial background in the atmosphere as several dominant taxa were found in independently conducted studies. Considering the results of Fahlgren et al. (2010) and Zweifel et al. (2012), our current results support this assumption. A similar hypothesis was postulated by DeLeon-Rodriguez et al. (2013) for higher altitudes in the upper troposphere. The main OTU present in 17% of all bacterial sequences was affiliated with the genus *Sphingomonas*, which was the dominant OTU in all samples. This bacterial genus is known to be ubiquitous in the environment and has subsequently been reported from marine and aquatic environments as well as from terrestrial habitats and plant root systems (Harrison et al. 2005; Fahlgren et al. 2010; Li et al. 2010; Zweifel et al. 2012). Overall bacterial diversity was highly diverse ranging from typical marine bacteria such as *Pseudoalteromonas* and marine sediment bacteria like *Andersienella* to plant-related taxa (*Rhizobium*,*Massilia*), soil-related bacteria such as *Hymenobacter*,*Curtobacterium*, and *Arthrobacter* and human-associated genera such as *Propionibacterium* and *Prevotella*. Furthermore, potential pathogenic genera including the Bacillus genera *Staphylococcus* and *Streptococcus* as well as potential ice nucleation bacteria such as *Pseudomonas* and *Pantoea* were found. Human-associated bacteria were also detected in other studies: Fahlgren et al. (2010) and Zweifel et al. (2012) found *Propionibacterium* in bioaerosols on coastal areas of Sweden and the boundary layer above the coastal areas of Sweden and Denmark. This would be a further indication for a wide atmospheric mixture as found by Li et al. (2010). However, members of the family Prevotellaceae were detected in aerosol samples at a high elevation site in Colorado, U.S.A. (Bowers et al. 2012). A broad occurrence of human-associated bacteria may be due to the influence of waste water plants as point sources for aerosolized bacteria to the atmosphere (Pascual et al. 2003; Karra and Katsivela 2007; Haas et al. 2010). Also potential ice condensation nuclei genera such as *Pantoea*,*Pseudomonas*,*Pedobacter*, and *Psychrobacter* have been found widely (Fahlgren et al. 2010; Bowers et al. 2012). The detected soil associated genera were also reported from other studies which dealt with urban aerosols (Fierer et al. 2008). The genus *Anderseniella* consists of a single species, *Anderseniella baltica*. Although it was isolated from sediments of the Baltic Sea (Brettar et al. 2007), it was not found in the studies of Fahlgren et al. (2010) and Zweifel et al. (2012). This is probably due to their cloning approach which resulted in fewer sequences compared to the high-throughput approach of the current study. The occurrence of marine sediment-associated bacteria may also underline the importance of beaches and/or coastal erosion processes for the formation of bioaerosols. Similar findings were made by Urbano et al. (2011) for the coastal area of San Diego (CA, USA).

Many bacteria, which occurred in high numbers in the present study were also dominant in other bioaerosol studies which investigated marine/coastal and terrestrial ecosystems. This is a further indication for a steady “bacterial core community” in the atmosphere, probably originating from strong area sources, for example, tropical rainforest, big grass fields, or soil areas and constantly getting distributed and mixed over long distances as stated by Gandolfi et al. (2013).

### Influence of environmental factors

The bacterial community was strongly affected by temperature, wind direction, and the sample location, whereas temperature and wind direction were negatively correlated. Only few studies identified environmental parameters which influence the structure of airborne microbial communities (Maron et al. 2006; Brodie et al. 2007; Bowers et al. 2012; Bertolini et al. 2013; Gandolfi et al. 2013). All these studies, however, conducted long-term sampling approaches at single (Maron et al. 2005, 2006; Bowers et al. 2012; Bertolini et al. 2013) or two sampling sites (Brodie et al. 2007). This clearly contrasts with the sampling design of the current investigation where the sampling events were conducted at different locations. All studies found temperature to be one main factor influencing the bacterial community composition. Temporal variability may be influenced by combined effects of differences in several meteorological factors, chemical composition, particulate matter, and the importance of the main source of PBAP rather than only temperature (Brodie et al. 2007; Bertolini et al. 2013). This is supported by our findings also for spatial variability. Temperature might be a function of wind direction, as samples were taken at different sites each featuring different temperatures. North winds were colder than southern winds which exhibit continental influence. The OTU sequencing results were, therefore, grouped into the wind directions, allowing for classification along a clearly defined system. The calculated backward trajectories revealed that several samples featured a stronger marine influence than the measured wind direction might anticipate. Although wind direction seems to be a good indicator it has to be regarded with caution as the days before sampling also need to be taken into account. Bacterial composition of marine bioaerosols seems to be influenced by several different environmental factors combined.

Additionally, differences in bacterial community composition among the sampling locations were affected by the sampling locality itself and their adjacent ecosystems. Similar studies for terrestrial environments revealed a strong influence of the adjacent land-use type (agricultural, rural, forest) on the airborne bacterial community and that the different sampling locations for microbial communities were significantly distinct from each other (Bowers et al. 2011a). This also seems to apply to marine/coastal environments. For example, the detected Cyanobacteria showed a distinct distributional pattern in our samples grouped by sampling locations. While the few sequences in the samples from the Skagerrak were mostly accounted to *Prochlorococcus*, sequences in the Baltic Sea mostly belonged to *Synechococcus*. The latter genus is a common Cyanobacterium present in the low salinity environment of the Baltic Sea (Partensky et al. 1999), particularly thriving in shallow water depths with increasing light intensities in the summer months (Jochem 1988). Therefore, these findings corroborate the general assumption that the bubble-bursting process of marine bioaerosol formation mostly transports bacteria on and near the sea surface, whereas bacteria from deeper parts of the water column have a much lower chance to get aerosolized. Similar conclusions were drawn by Cho and Hwang (2011) who compared airborne bacteria with bacteria from the sea surface at the same sampling location. Aller et al. (2005) also identified the sea surface microlayer as a source for marine bioaerosols.

The bacterial distribution was affected by different environmental factors (wind direction, sampling location) and this effect could already be seen on the phylum level. For example, Firmicutes bacteria were detected at all sampling locations but were nearly absent in eastern and southeastern winds. On class level, Gammaproteobacteria were present in every wind direction and location but showed a higher proportion of the sequences in southern wind directions where it was the dominant class. In the class Alphaproteobacteria, the plant/root-associated Rhizobiaceae (genus Rhizobium) were only present in “E,” “SE,” “S,” and “SW” sampling direction, but were found at all sampling locations except “SK,” where only northern and northwest winds were detected.

In contrast, Betaproteobacteria were evenly distributed among the different wind directions but were disproportionally more abundant in samples from “SK.” A similar picture is present for the phylum Actinobacteria with the classes Actinobacteria and Acidimicrobia, where in “SK” samples Acidimicrobia represented 31% (uncultured Acidimicrobiaceae) of all Actinobacteria sequences while it was below the 1% threshold at other sampling locations.

In summary, we found that a small number of bacterial OTUs made up more than 75% of all bacterial sequences. The most abundant OTU belonging to the genus *Sphingomona*s has also been reported in other bioaerosol studies from many different environments (urban, rural, forest, high alpine). This further supports the assumption of a bacterial core community in the atmosphere, released from strong area aerosolizing sources and then getting mixed and dispersed over long distances. The most abundant bacterial genera are associated with different places of origin: marine (*Pseudoalteromonas*), marine sediment (*Anderseniella*), plant/root (*Rhizobium*), human (*Propionibacterium*), potential pathogens (*Staphylococcus*), soil (*Hymenobacter*). This highly diverse airborne bacterial community suggests the existence of several heterogeneous sources for bioaerosols of/near marine and coastal environments. The two most important environmental parameters were temperature as a function of wind direction and the sampling location. Some bacteria were found at all locations but were absent in some wind directions and *vice versa*. The wind direction can be a good indicator for possible influences (marine, continental) but additionally backward trajectories are needed for detailed information.

DNA yield after DNA extraction was comparably low. The combination of low DNA yield and high diversity contributed to rarefaction curves that did not reach a plateau. Thus, the chosen sampling duration limits the breadth of the conclusions that can be drawn, as not the whole bacterial community may have been accessed. Further clarification of sampling durations and sampling method evaluation for marine bioaerosols is needed to strengthen future investigations in that field.

## Supporting Information

**Table S1.**Sample classifications for the different alpha- and betadiversity testing groups. **Table S2.** Sampling Date, area, time, exact position, and measured environmental parameters. **Table S3.** PERMANOVA pairwise test for the factor “sampling area” based on Bray–Curtis dissimilarities of OTUs (16S amplicon sequencing). **Table S4.** PERMANOVA pairwise test for the factor “cardinal direction” group based on Bray–Curtis dissimilarities of OTUs (16S amplicon sequencing). **Table S5.** PERMANOVA pairwise test for wind direction group based on Bray–Curtis dissimilarities of OTUs (16S amplicon sequencing). **Table S6.** PERMANOVA pairwise test for rain group based on Bray–Curtis dissimilarities of OTUs (16S amplicon sequencing). **Table S7.** Principal components analysis (PCA) calculated eigenvalues. Displayed are the eigenvalues for the first five axes including the percentage of variation explained by each axis and the accumulation of them. **Table S8.** Result of the 16S gene amplicon sequencing. Sequences obtained per sample, percentage of chloroplast and mitochondrial sequences, and sequences and percentage of sequences which entered further analyses. **Table S9.** List of the 37 most abundant OTUs with classification, total number of sequences, and sequences partitioned by wind direction and sampling location. **Figure S1.** Principal coordinates analysis (PCO) of the pairwise distances between bacterial communities between the five different sampling locations as calculated using Bray–Curtis dissimilarities. **Figure S2.** Principal components analysis (PCA) of the pairwise distances of environmental variables measured for the samples calculated using the Euclidean distances. **Figure S3.** Taxonomic classification of Betaproteobacteria reads grouped in wind direction and sampling location on family level using SILVA classifier based on 98% similarity omitting singletons (*n* = 1) and rare reads (<1%). The amount of percentage proportion contribution of each family per group is indicated by color of cell; darker color represents higher contribution. **Figure S4.** Taxonomic classification of Gammaproteobacteria reads grouped in wind direction and sampling direction on family level using SILVA classifier based on 98% similarity omitting singletons (*n* = 1) and rare reads (<1%). The amount of percentage proportion contribution of each family per group is indicated by color of cell; darker color represents higher contribution. **Figure S5.** Taxonomic classification of Firmicutes reads grouped in wind direction and sampling location on class level using SILVA classifier based on 98% similarity omitting singletons (*n* = 1) and rare reads (<1%). The amount of percentage proportion contribution of each class per group is indicated by color of cell; darker color represents higher contribution. **Figure S6.** Taxonomic classification of Clostridia reads grouped in wind direction and sampling location on family level using SILVA classifier based on 98% similarity omitting singletons (*n* = 1) and rare reads (<1%). The amount of percentage proportion contribution of each family per group is indicated by color of cell; darker color represents higher contribution. **Figure S7.** Taxonomic classification of Bacilli reads grouped in wind direction and sampling location on family level using SILVA classifier based on 98% similarity omitting singletons (*n* = 1) and rare reads (<1%). The amount of percentage proportion contribution of each family per group is indicated by color of cell; darker color represents higher contribution. **Figure S8.** Taxonomic classification of Bacteroidetes reads grouped in wind direction and sampling location on class level using SILVA classifier based on 98% similarity omitting singletons (*n* = 1) and rare reads (<1%). The amount of percentage proportion contribution of each class per group is indicated by color of cell; darker color represents higher contribution. **Figure S9.** Taxonomic classification of Bacteroidetes reads grouped in wind direction and sampling location on family level using SILVA classifier based on 98% similarity omitting singletons (*n* = 1) and rare reads (<1%). The amount of percentage proportion contribution of each family per group is indicated by color of cell; darker color represents higher contribution.
