# Supplementary material for: Spatial distribution of marine airborne bacterial communities
Source: Microbiologyopen. 2015 Mar 19;4(3):475–90. doi: 10.1002/mbo3.253 (PMC4475389; doi:10.1002/mbo3.253)
Supplement: Supplementary file 1 — Table S1.Sample classifications for the different alpha- and betadiversity testing groups. Table S2. Sampling Date, area, time, exact position, and measured environmental parameters. Table S3. PERMANOVA pairwise test for the factor “sampling area” based on Bray–Curtis dissimilarities of OTUs (16S amplicon sequencing). Table S4. PERMANOVA pairwise test for the factor “cardinal direction” group based on Bray–Curtis dissimilarities of OTUs (16S amplicon sequencing). Table S5. PERMANOVA pairwise test for wind direction group based on Bray–Curtis dissimilarities of OTUs (16S amplicon sequencing). Table S6. PERMANOVA pairwise test for rain group based on Bray–Curtis dissimilarities of OTUs (16S amplicon sequencing). Table S7. Principal components analysis (PCA) calculated eigenvalues. Displayed are the eigenvalues for the first five axes including the percentage of variation explained by each axis and the accumulation of them. Table S8. Result of the 16S gene amplicon sequencing. Sequences obtained per sample, percentage of chloroplast and mitochondrial sequences, and sequences and percentage of sequences which entered further analyses. Table S9. List of the 37 most abundant OTUs with classification, total number of sequences, and sequences partitioned by wind direction and sampling location. Figure S1. Principal coordinates analysis (PCO) of the pairwise distances between bacterial communities between the five different sampling locations as calculated using Bray–Curtis dissimilarities. Figure S2. Principal components analysis (PCA) of the pairwise distances of environmental variables measured for the samples calculated using the Euclidean distances. Figure S3. Taxonomic classification of Betaproteobacteria reads grouped in wind direction and sampling location on family level using SILVA classifier based on 98% similarity omitting singletons (n = 1) and rare reads (<1%). The amount of percentage proportion contribution of each family per group is indicated by color of ce [file mbo30004-0475-sd1.doc]

Supplement Material

Table S1. Sample classifications for the different alpha- and betadiversity testing groups.

| Sample | Sample Area | BWT Influence | BWT | Cardinal direction | Wind direction | Height | Rain |
| --- | --- | --- | --- | --- | --- | --- | --- |
| H01 | North Sea | mixed | crossing, no high altitude | W | SW | low | n |
| H02 | North Sea | mixed | no crossing, no high altitude | W | W | low | n |
| H03 | North Sea | mixed | no crossing, no high altitude | W | W | low | n |
| H04 | North Sea | mixed | no crossing, no high altitude | W | NW | low | n |
| H06 | North Sea | marine | no crossing, no high altitude | N | NW | 1000 | n |
| H08 | North Sea | more marine | no crossing, no high altitude | N | N | low | n |
| H11 | Skagerrak | marine | crossing, high altitude | W | NW | 1500 | 0 |
| H12 | Skagerrak | marine | crossing, no high altitude | N | NW | 500 | 0 |
| H13 | Kattegat | more marine | no crossing, no high altitude | W | W | 500 | n |
| H14 | Kattegat | marine | no crossing, no high altitude | W | W | low | n |
| H15 | Kattegat | more marine | no crossing, no high altitude | W | W | low | n |
| H16 | Kattegat | more marine | no crossing, no high altitude | W | SW | low | n |
| H17 | Kattegat | mixed | no crossing, no high altitude | S | S | 1000 | 60 |
| H18 | Kattegat | mixed | no crossing, no high altitude | S | S | 1000 | 60 |
| H19 | Kattegat | mixed | no crossing, high altitude | S | SE | 2000 | 60 |
| H20 | Kattegat | mixed | no crossing, no high altitude | S | SE | 500 | n |
| H21 | Baltic Sea | continental | no crossing, no high altitude | W | W | low | 6 |
| H22 | Baltic Sea | more continental | no crossing, no high altitude | W | NW | low | 6 |
| H23 | Baltic Sea | more continental | no crossing, no high altitude | W | NW | low | 6 |
| H24 | Baltic Sea | more continental | no crossing, no high altitude | W | W | low | 6 |
| H25 | Baltic Sea | mixed | no crossing, no high altitude | E | SE | low | n |
| H26 | Baltic Sea | mixed | no crossing, no high altitude | E | E | low | n |
| H28 | Baltic Sea | mixed | no crossing, no high altitude | E | E | low | n |
| H29 | Baltic Sea | continental | no crossing, no high altitude | W | SW | low | 60 |
| H30 | Baltic Sea | continental | no crossing, no high altitude | W | SW | low | 60 |
| H31 | Baltic Sea | continental | no crossing, no high altitude | W | W | low | n |
| H32 | Baltic Sea | continental | no crossing, no high altitude | E | E | low | 6 |
| H33 | Baltic Sea | more continental | no crossing, no high altitude | W | SW | low | 6 |
| H34 | Kiel Canal | more continental | crossing, no high altitude | W | SW | 500 | 60 |
| H35 | Kiel Canal | more continental | crossing, no high altitude | S | S | low | n |
| H36 | Kiel Canal | more continental | crossing, no high altitude | S | S | low | 60 |

Table S2. Sampling Date, area, time, exact position and measured environmental parameters.

| Sample | Date | Geography | UTC | Long | Lat | global radiation | longwave radiation | Absolute wind direction | Absolute wind speed | Air pressure | Air temperature | Humidity |
| --- | --- | --- | --- | --- | --- | --- | --- | --- | --- | --- | --- | --- |
|  |  |  |  |  |  | W/m² | W/m² | deg | m/s | hpa | °C | % |
| H01 | August 17, 2011 | North Sea | 06:45-07:45 | 54.588667 | 08.250000 | 223.9 | 344.4 | 228.7 | 7.8 | 1014.2 | 16.9 | 89.7 |
| H02 | August 17, 2011 | North Sea | 10:40-11:45 | 54.579833 | 8.055667 | 697.1 | 311.5 | 274.4 | 8.5 | 1014.8 | 16.6 | 82.4 |
| H03 | August 17, 2011 | North Sea | 11:50-12:50 | 54.699 | 8.003167 | 746.1 | 299.6 | 267.4 | 8.4 | 1014.9 | 16.1 | 72.7 |
| H04 | August 18, 2011 | North Sea | 06:30-07:35 | 55.307167 | 7.787833 | 270.5 | 327.9 | 293.6 | 5.1 | 1016.6 | 15.7 | 82.9 |
| H05 | August 18, 2011 | North Sea | 07:40-08:45 | 55.461 | 7.562833 | 376.1 | 329.3 | 294.2 | 5.2 | 1016.8 | 15.8 | 82.9 |
| H06 | August 18, 2011 | North Sea | 11:00-12:05 | 55.7845 | 7.670333 | 647.4 | 319.7 | 328.2 | 3.8 | 1017.1 | 15.9 | 82.3 |
| H07 | August 18, 2011 | North Sea | 12:10-13:10 | 55.891167 | 7.68 | 644.3 | 304.2 | 335.9 | 3.8 | 1016.7 | 15.9 | 79.9 |
| H08 | August 18, 2011 | North Sea | 15:55-17:00 | 56.1275 | 7.702167 | 306.3 | 294.7 | 350.4 | 3.2 | 1015.3 | 15.9 | 79.3 |
| H09 | August 19, 2011 | North Sea | 06:55-08:00 | 56.993167 | 8.031167 | 52.3 | 348.8 | 58.3 | 4.5 | 1007.9 | 13.3 | 81.6 |
| H10 | August 19, 2011 | North Sea | 08:05-09:10 | 57.1445 | 8.329667 | 91.6 | 350.9 | 315.1 | 5.2 | 1007.9 | 13.2 | 84.2 |
| H11 | August 19, 2011 | Skagerrak | 12:50-13:55 | 57.522 | 9.3825 | 161.0 | 355.2 | 297.1 | 7.8 | 1006.3 | 13.9 | 84.3 |
| H12 | August 19, 2011 | Skagerrak | 14:00-15:05 | 57.672 | 9.639333 | 144.7 | 357.3 | 315.7 | 10.1 | 1005.8 | 14.6 | 83.3 |
| H13 | August 20, 2011 | Kattegat | 06:55-08:00 | 57.660833 | 11.051500 | 439.6 | 289.7 | 284.3 | 15.2 | 1012.8 | 15.8 | 72.9 |
| H14 | August 20, 2011 | Kattegat | 08:05-09:05 | 57.4835 | 11.2465 | 541.6 | 295.2 | 283.9 | 13.9 | 1013.9 | 15.9 | 74.9 |
| H15 | August 20, 2011 | Kattegat | 12:00-13:00 | 56.9265 | 11.438833 | 636.7 | 314.7 | 251.8 | 5.0 | 1016.0 | 17.2 | 72.5 |
| H16 | August 20, 2011 | Kattegat | 13:05-14:10 | 56.865333 | 11.111333 | 316.7 | 341.4 | 227.2 | 4.2 | 1015.9 | 17.9 | 74.7 |
| H17 | August 21, 2011 | Kattegat | 06:45-07:50 | 56.7935 | 11.03 | 191.7 | 347.6 | 185.3 | 5.8 | 1017.1 | 15.7 | 89.4 |
| H18 | August 21, 2011 | Kattegat | 07:55-08:55 | 56.655333 | 11.1595 | 317.7 | 321.0 | 176.5 | 7.2 | 1016.7 | 16.5 | 87.1 |
| H19 | August 21, 2011 | Kattegat | 11:15-12:20 | 56.4845 | 11.536833 | 568.1 | 322.3 | 136.9 | 5.6 | 1015.7 | 17.9 | 85.9 |
| H20 | August 21, 2011 | Kattegat | 14:10-15:15 | 56.461667 | 11.998 | 228.9 | 341.6 | 145.0 | 9.1 | 1014.1 | 19.4 | 73.9 |
| H21 | August 22, 2011 | Baltic Sea | 06:50-07:55 | 54.759833 | 13.067167 | 91.6 | 378.9 | 288.9 | 8.5 | 1013.3 | 17.2 | 96.9 |
| H22 | August 22, 2011 | Baltic Sea | 08:00-09:05 | 54.7145 | 12.975167 | 299.4 | 353.4 | 296.5 | 8.9 | 1014.2 | 17.8 | 95.0 |
| H23 | August 22, 2011 | Baltic Sea | 11:30-12:35 | 54.668 | 12.783333 | 730.3 | 303.4 | 297.4 | 7.8 | 1016.9 | 16.9 | 81.6 |
| H24 | August 22, 2011 | Baltic Sea | 12:40-13:40 | 54.644833 | 12.5565 | 606.4 | 311.4 | 291.9 | 4.5 | 1017.7 | 17.8 | 79.1 |
| H25 | August 23, 2011 | Baltic Sea | 06:45-07:50 | 54.321833 | 11.886333 | 182.2 | 344.0 | 130.0 | 3.3 | 1020.7 | 16.9 | 90.8 |
| H26 | August 23, 2011 | Baltic Sea | 07:55-09:00 | 54.2995 | 11.7695 | 180.1 | 359.2 | 96.3 | 4.9 | 1019.9 | 17.6 | 83.3 |
| H27 | August 23, 2011 | Baltic Sea | 11:35-12:40 | 54.234333 | 11.4525 | 581.3 | 338.8 | 103.9 | 5.9 | 1018.5 | 18.2 | 81.1 |
| H28 | August 23, 2011 | Baltic Sea | 12:45-13:45 | 54.1635 | 11.254 | 531.1 | 333.1 | 86.0 | 6.1 | 1017.5 | 18.8 | 79.6 |
| H29 | August 24, 2011 | Baltic Sea | 06:40-07:45 | 54.517667 | 10.845167 | 197.8 | 371.6 | 241.8 | 3.8 | 1013.4 | 17.9 | 93.2 |
| H30 | August 24, 2011 | Baltic Sea | 07:50-08:50 | 54.489833 | 10.808667 | 331.3 | 359.7 | 226.2 | 2.3 | 1013.8 | 18.3 | 93.7 |
| H31 | August 24, 2011 | Baltic Sea | 12:15-13:20 | 54.621167 | 10.422833 | 452.0 | 366.0 | 250.2 | 2.8 | 1013.8 | 19.8 | 85.0 |
| H32 | August 24, 2011 | Baltic Sea | 16:25-17:30 | 54.747667 | 10.112833 | 91.4 | 342.1 | 91.1 | 4.4 | 1013.4 | 19.0 | 92.1 |
| H33 | August 25, 2011 | Baltic Sea | 07:50-08:55 | 54.510667 | 10.1175 | 304.9 | 363.7 | 261.7 | 4.1 | 1015.2 | 16.1 | 96.1 |
| H34 | August 25, 2011 | Kiel Canal | 11:45-12:50 | 54.389 | 10.1945 | 483.4 | 357.4 | 232.5 | 2.9 | 1015.4 | 18.8 | 78.9 |
| H35 | August 25, 2011 | Kiel Canal | 12:55-13:55 | 54.361333 | 10.061667 | 582.8 | 340.3 | 196.9 | 1.3 | 1015.2 | 20.4 | 72.4 |
| H36 | August 25, 2011 | Kiel Canal | 14:00-15:00 | 54.362833 | 9.859 | 392.8 | 339.8 | 177.9 | 1.2 | 1014.8 | 21.4 | 69.2 |

Table S3. PERMANOVA pair wise test for the factor ‘sampling area’ based on Bray-Curtis dissimilarities of OTUs (16S amplicon sequencing).

| Groups | t (perm) | p (perm)^1^ |
| --- | --- | --- |
| North Sea, Skagerrak | 1.0615 | 0.197 |
| North Sea, Kattegat | 0.96675 | 0.462 |
| North Sea, Baltic Sea | 1.3509 | **0.049** |
| North Sea, Kiel Canal | 1.2275 | 0.121 |
| Skagerrak, Kattegat | 1.1613 | 0.175 |
| Skagerrak, Baltic Sea | 1.4988 | **0.0437*** |
| Skagerrak, Kiel Canal | 1.8003 | 0.096 |
| Kattegat, Baltic Sea | 0.99075 | 0.404 |
| Kattegat, Kiel Canal | 1.0648 | 0.288 |
| Baltic Sea, Kiel Canal | 1.0376 | 0.267 |

1: Significant results (*p (perm)*<0.05) are highlighted in bold

Displayed are pair-wise *a posteriori* comparisons of the factor ‘sampling area’ with at least 100 unique permutations per comparison. Below 100 unique permutations the Monte Carlo permutation was used (*).

Table S4. PERMANOVA pair-wise test for the factor ‘cardinal direction’ group based on Bray-Curtis dissimilarities of OTUs (16S amplicon sequencing).

| Groups | t (perm) | p (perm)^1^ |
| --- | --- | --- |
| W, N | 1.915 | 0.106 |
| W, S | 0.99067 | 0.441 |
| W, E | 1.3059 | **0.043** |
| N, S | 1.2778 | 0.111 |
| N, E | 1.7473 | **0.0411*** |
| S, E | 1.1389 | 0.223 |

1: Significant results (*p (perm)*<0.05) are highlighted in bold

Displayed are pair-wise *a posteriori* comparisons of the factor ‘cardinal direction with at least 100 unique permutations per comparison. Below 100 unique permutations the Monte Carlo permutation was used (*)

Table S5. PERMANOVA pair-wise test for wind direction group based on Bray-Curtis dissimilarities of OTUs (16S amplicon sequencing).

| Groups | t (perm) | p (perm)^1^ |
| --- | --- | --- |
| SW, W | 1.5497 | **0.01** |
| SW, NW | 1.6776 | **0.002** |
| SW, N | 1.7433 | 0.149 |
| SW, S | 1.2842 | 0.057 |
| SW, SE | 1.0953 | 0.245 |
| SW, E | 1.0935 | 0.21 |
| W, NW | 1.0597 | 0.246 |
| W, N | 1.0127 | 0.568 |
| W, S | 1.1625 | 0.133 |
| W, SE | 1.2366 | 0.064 |
| W, E | 1.4072 | **0.031** |
| NW, N | 0.86427 | 1 |
| NW, S | 1.1162 | 0.14 |
| NW, SE | 1.3704 | 0.1096* |
| NW, E | 1.4835 | 0.0666* |
| N, S | 1.1725 | 0.181 |
| N, SE | 1.7674 | 0.234 |
| N, E | 1.9946 | 0.23 |
| S, SE | 1.2403 | 0.146 |
| S, E | 1.3476 | 0.118 |
| SE, E | 0.87567 | 0.731 |

1: Significant results (*p (perm)*<0.05) are highlighted in bold

Displayed are pair-wise *a posteriori* comparisons of the factor ‘wind direction’ with at least 100 unique permutations per comparison. Below 100 unique permutations the Monte Carlo permutation was used (*).

Table S6. PERMANOVA pair-wise test for rain group based on Bray-Curtis dissimilarities of OTUs (16S amplicon sequencing).

| Groups | T(perm) | P(perm) |
| --- | --- | --- |
| n, 0 | 1.3498 | 0.051 |
| n, 60 | 1.1376 | 0.164 |
| n, 6 | 1.2004 | 0.113 |
| 0, 60 | 1.4160 | 0.061 |
| 0, 6 | 1.1806 | 0.144 |
| 60, 6 | 0.97765 | 0.421 |

Displayed are pair-wise *a posteriori* comparisons of the factor ‘rain with at least 100 unique permutations per comparison.

Table S7. Principal components analysis (PCA) calculated eigenvalues. Displayed are the eigenvalues for the first five axes including the percentage of variation explained by each axis and the accumulation of them.

| PC | Eigenvalues | %Variation | Cum.%Variation |
| --- | --- | --- | --- |
| 1 | 2.89 | 32.1 | 32.1 |
| 2 | 2.30 | 25.6 | 57.7 |
| 3 | 1.28 | 14.2 | 71.9 |
| 4 | 0.925 | 10.3 | 82.2 |
| 5 | 0.708 | 7.9 | 90 |

Table S8. Result of the 16S gene amplicon sequencing. Sequences obtained per sample, percentage of chloroplast and mitochondrial sequences and sequences and percentage of sequences which entered further analyses.

| Sample | Date | Number of Sequences before trimming | Sequences after trimming | | Mitochondrial sequences (%) | Chloroplast sequences (%) | Sequences left after trimming (%) |
| --- | --- | --- | --- | --- | --- | --- | --- |
| H01 | August 17, 2011 | 12507 | | 11537 | 0.4 | 7.1 | 92.2 |
| H02 | August 17, 2011 | 8518 | | 7259 | 2.9 | 11.7 | 85.2 |
| H03 | August 17, 2011 | 1642 | | 1435 | 0.1 | 12.4 | 87.4 |
| H04 | August 18, 2011 | 1111 | | 483 | 0.5 | 55.9 | 43.5 |
| H06 | August 18, 2011 | 3968 | | 3670 | 0.5 | 6.8 | 92.5 |
| H08 | August 18, 2011 | 6744 | | 756 | 0.3 | 88.5 | 11.2 |
| H11 | August 19, 2011 | 2682 | | 2116 | 1.4 | 19.7 | 78.9 |
| H12 | August 19, 2011 | 8139 | | 2829 | 0.1 | 65.1 | 34.8 |
| H13 | August 20, 2011 | 6895 | | 1765 | 0.9 | 73.5 | 25.6 |
| H14 | August 20, 2011 | 5270 | | 1557 | 1.6 | 68.9 | 29.5 |
| H15 | August 20, 2011 | 5516 | | 1533 | 26.1 | 46.1 | 27.8 |
| H16 | August 20, 2011 | 10250 | | 5144 | 23.5 | 26.2 | 50.2 |
| H17 | August 21, 2011 | 8750 | | 8537 | 0.1 | 2.2 | 97.6 |
| H18 | August 21, 2011 | 5041 | | 4900 | 0.1 | 2.7 | 97.2 |
| H19 | August 21, 2011 | 19233 | | 2627 | 69.2 | 17.2 | 13.7 |
| H20 | August 21, 2011 | 7451 | | 5723 | 0.6 | 22.5 | 76.8 |
| H21 | August 22, 2011 | 3934 | | 3812 | 0.2 | 2.9 | 96.9 |
| H22 | August 22, 2011 | 7414 | | 6852 | 0.4 | 7.1 | 92.4 |
| H23 | August 22, 2011 | 22865 | | 1239 | 58.0 | 36.6 | 5.4 |
| H24 | August 22, 2011 | 13687 | | 2299 | 37.7 | 45.5 | 16.8 |
| H25 | August 23, 2011 | 11582 | | 4832 | 0.1 | 58.2 | 41.7 |
| H26 | August 23, 2011 | 10864 | | 2568 | 8.0 | 68.3 | 23.6 |
| H28 | August 23, 2011 | 15153 | | 6953 | 32.9 | 21.2 | 45.9 |
| H29 | August 24, 2011 | 7663 | | 2840 | 4.3 | 58.6 | 37.1 |
| H30 | August 24, 2011 | 11404 | | 3570 | 0.2 | 68.5 | 31.3 |
| H31 | August 24, 2011 | 6405 | | 1826 | 17.9 | 53.6 | 28.5 |
| H32 | August 24, 2011 | 12239 | | 6771 | 0.9 | 43.7 | 55.3 |
| H33 | August 25, 2011 | 5424 | | 2517 | 32.1 | 21.4 | 46.4 |
| H34 | August 25, 2011 | 15228 | | 2347 | 8.6 | 76.0 | 15.4 |
| H35 | August 25, 2011 | 23327 | | 3295 | 9.5 | 76.4 | 14.1 |
| H36 | August 25, 2011 | 12075 | | 3632 | 5.4 | 64.5 | 30.1 |

Table S9. List of the 37 most abundant OTUs with classification, total number of sequences and sequences partitioned by wind direction and sampling location.

| OTU | Phylum | Class | Family | Genus | Sequences | Wind direction | | | | | | | | Sampling location | | | | |
| --- | --- | --- | --- | --- | --- | --- | --- | --- | --- | --- | --- | --- | --- | --- | --- | --- | --- | --- |
|  |  |  |  |  |  | N | E | SE | S | SW | W | NW | NS | | SK | KT | BS | KC |
| 37 | Actinobacteria | Actinobacteria | Corynebacteriales, uncult. | Corynebacteriales, uncult. | 580 | 0 | 188 | 49 | 61 | 198 | 59 | 25 | 137 | | 5 | 137 | 235 | 66 |
| 82 | Actinobacteria | Actinobacteria | Microbacteriaceae | unident. Microbacteriaceae | 2809 | 1 | 295 | 263 | 94 | 1316 | 782 | 58 | 1217 | | 18 | 827 | 642 | 105 |
| 90 | Actinobacteria | Actinobacteria | Microbacteriaceae | Curtobacterium | 2820 | 5 | 478 | 567 | 218 | 1093 | 424 | 34 | 698 | | 8 | 586 | 1328 | 199 |
| 99 | Actinobacteria | Actinobacteria | Microbacteriaceae | Microbacterium | 572 | 1 | 96 | 92 | 49 | 233 | 71 | 30 | 162 | | 5 | 173 | 194 | 38 |
| 113 | Actinobacteria | Actinobacteria | Micrococcaceae | Arthrobacter | 2608 | 5 | 105 | 69 | 1664 | 463 | 236 | 65 | 461 | | 17 | 1678 | 259 | 192 |
| 132 | Actinobacteria | Actinobacteria | Propionibacteriaceae | Friedmanniella | 753 | 1 | 19 | 7 | 9 | 36 | 11 | 670 | 681 | | 1 | 19 | 43 | 9 |
| 136 | Actinobacteria | Actinobacteria | Propionibacteriaceae | Propionibacterium | 2819 | 87 | 43 | 81 | 1242 | 140 | 495 | 731 | 262 | | 272 | 1362 | 859 | 64 |
| 170 | Bacteroidetes | Bacteroidia | Prevotellaceae | Prevotella | 2791 | 58 | 241 | 61 | 734 | 311 | 560 | 826 | 733 | | 260 | 853 | 893 | 52 |
| 180 | Bacteroidetes | Cytophagia | Cytophagaceae | Dyadobacter | 1145 | 0 | 299 | 301 | 137 | 248 | 100 | 60 | 60 | | 0 | 308 | 626 | 151 |
| 183 | Bacteroidetes | Cytophagia | Cytophagaceae | Hymenobacter | 5486 | 4 | 1342 | 433 | 265 | 1721 | 1278 | 442 | 2471 | | 72 | 703 | 1857 | 382 |
| 190 | Bacteroidetes | Cytophagia | Cytophagaceae | Spirosoma | 1085 | 0 | 361 | 130 | 94 | 298 | 85 | 117 | 146 | | 1 | 127 | 682 | 129 |
| 192 | Bacteroidetes | Cytophagia | Cytophagaceae | Cytophagaceae, uncult. | 747 | 0 | 43 | 65 | 20 | 55 | 554 | 10 | 16 | | 1 | 559 | 149 | 22 |
| 198 | Bacteroidetes | Flavobacteria | Cryomorphaceae | Brumimicrobium | 2072 | 0 | 9 | 3 | 2005 | 27 | 12 | 16 | 6 | | 6 | 1968 | 26 | 66 |
| 206 | Bacteroidetes | Flavobacteria | Flavobacteriaceae | Chryseobacterium | 2085 | 7 | 533 | 475 | 173 | 570 | 111 | 215 | 410 | | 24 | 488 | 965 | 197 |
| 210 | Bacteroidetes | Flavobacteria | Flavobacteriaceae | Flavobacterium | 568 | 0 | 169 | 100 | 52 | 90 | 123 | 34 | 132 | | 2 | 139 | 235 | 60 |
| 222 | Bacteroidetes | Flavobacteria | Flavobacteriaceae | Sufflavibacter | 643 | 1 | 181 | 32 | 43 | 255 | 114 | 17 | 260 | | 10 | 100 | 246 | 27 |
| 225 | Bacteroidetes | Flavobacteria | Flavobacteriaceae | Winogradskyella | 639 | 0 | 3 | 1 | 615 | 11 | 7 | 2 | 3 | | 1 | 581 | 10 | 44 |
| 244 | Bacteroidetes | Sphingobacteriia | Sphingobacteriaceae | Pedobacter | 6452 | 5 | 1661 | 921 | 441 | 995 | 252 | 2177 | 371 | | 20 | 1016 | 4578 | 467 |
| 278 | Cyanobacteria | Cyanobacteria | FamilyI | Synechococcus | 1347 | 5 | 12 | 23 | 22 | 39 | 266 | 980 | 26 | | 39 | 88 | 1167 | 27 |
| 322 | Firmicutes | Bacilli | Staphylococcaceae | Staphylococcus | 4367 | 113 | 20 | 98 | 795 | 1050 | 1294 | 997 | 397 | | 313 | 932 | 2573 | 152 |
| 344 | Firmicutes | Bacilli | Streptococcaceae | Streptococcus | 963 | 3 | 8 | 19 | 276 | 30 | 385 | 242 | 55 | | 38 | 279 | 574 | 17 |
| 346 | Firmicutes | Clostridia | Clostridiaceae | Clostridium | 772 | 7 | 31 | 32 | 213 | 150 | 112 | 227 | 81 | | 121 | 239 | 290 | 41 |
| 454 | Proteobacteria | Alphaproteobacteria | Methylobacteriaceae | Methylobacterium | 922 | 5 | 187 | 175 | 58 | 322 | 69 | 106 | 186 | | 15 | 198 | 452 | 71 |
| 467 | Proteobacteria | Alphaproteobacteria | Rhizobiaceae | Rhizobium | 3937 | 4 | 1337 | 1075 | 329 | 881 | 244 | 67 | 584 | | 7 | 1088 | 1932 | 326 |
| 469 | Proteobacteria | Alphaproteobacteria | Rhodobiaceae | Anderseniella | 4535 | 102 | 593 | 938 | 851 | 1130 | 677 | 243 | 160 | | 24 | 595 | 2616 | 1139 |
| 545 | Proteobacteria | Alphaproteobacteria | Sphingomonadaceae | Sphingomonas | 20194 | 68 | 3192 | 3520 | 689 | 6571 | 5036 | 1116 | 7318 | | 155 | 5527 | 6410 | 782 |
| 579 | Proteobacteria | Betaproteobacteria | Comamonadaceae | Variovorax | 547 | 1 | 117 | 52 | 55 | 204 | 58 | 60 | 157 | | 11 | 80 | 235 | 64 |
| 586 | Proteobacteria | Betaproteobacteria | Oxalobacteraceae | Massilia | 3319 | 4 | 571 | 348 | 144 | 1034 | 358 | 860 | 911 | | 787 | 357 | 1114 | 150 |
| 587 | Proteobacteria | Betaproteobacteria | Oxalobacteraceae | Oxalobacter | 929 | 16 | 58 | 30 | 315 | 366 | 98 | 46 | 30 | | 2 | 165 | 348 | 384 |
| 598 | Proteobacteria | Betaproteobacteria | Neisseriaceae | Neisseriaceae, uncult. | 823 | 13 | 22 | 32 | 49 | 43 | 101 | 563 | 27 | | 31 | 61 | 687 | 17 |
| 651 | Proteobacteria | Gammaproteobacteria | Pseudoalteromonadaceae | Pseudoalteromonas | 3280 | 2 | 33 | 45 | 2631 | 288 | 212 | 69 | 47 | | 16 | 2352 | 307 | 558 |
| 667 | Proteobacteria | Gammaproteobacteria | Enterobacteriaceae | Pantoea | 556 | 0 | 220 | 63 | 79 | 144 | 38 | 12 | 74 | | 2 | 52 | 350 | 78 |
| 690 | Proteobacteria | Gammaproteobacteria | Oceanospirillaceae | Marinomonas | 544 | 1 | 5 | 3 | 475 | 13 | 33 | 14 | 17 | | 2 | 473 | 37 | 15 |
| 700 | Proteobacteria | Gammaproteobacteria | Moraxellaceae | Acinetobacter | 633 | 10 | 84 | 18 | 78 | 251 | 76 | 116 | 125 | | 19 | 66 | 383 | 40 |
| 704 | Proteobacteria | Gammaproteobacteria | Moraxellaceae | Psychrobacter | 3277 | 0 | 28 | 41 | 739 | 91 | 2216 | 162 | 726 | | 20 | 688 | 1716 | 127 |
| 707 | Proteobacteria | Gammaproteobacteria | Pseudomonadaceae | Pseudomonas | 1827 | 9 | 414 | 294 | 228 | 351 | 238 | 293 | 268 | | 170 | 313 | 901 | 175 |
| 709 | Proteobacteria | Gammaproteobacteria | Salinisphaeraceae | Salinisphaera | 546 | 0 | 4 | 4 | 6 | 17 | 510 | 5 | 519 | | 3 | 7 | 15 | 2 |


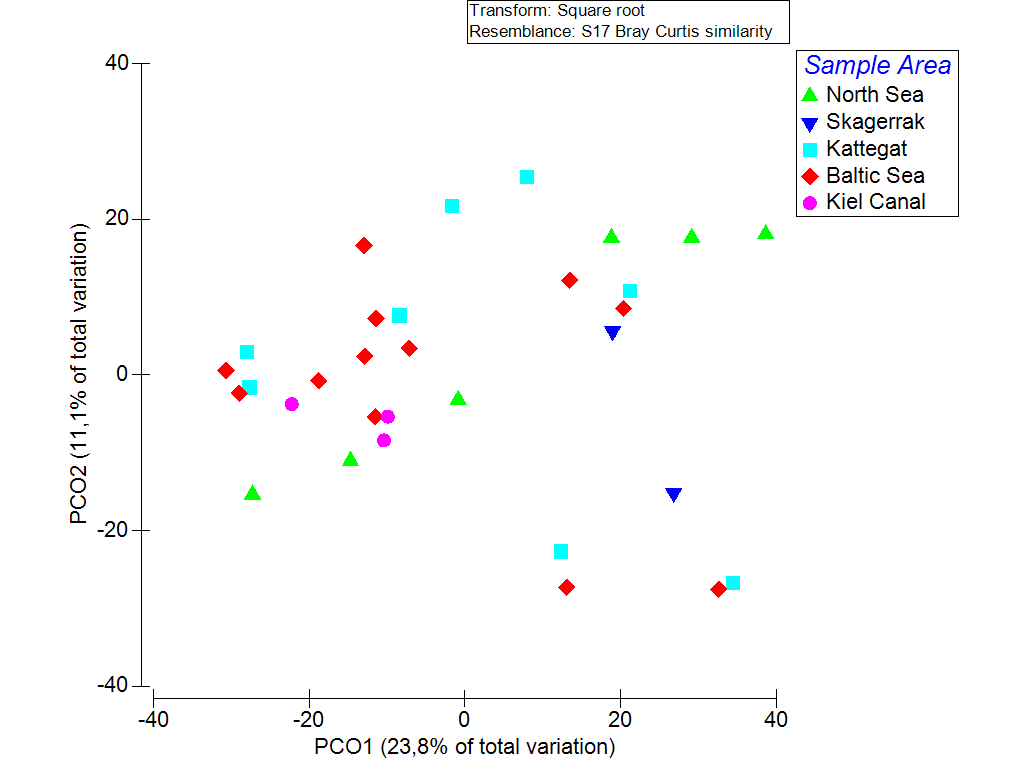


Fig. S1 Principal coordinates analysis (PCO) of the pair-wise distances between bacterial communities between the five different sampling locations as calculated using Bray-Curtis dissimilarities.


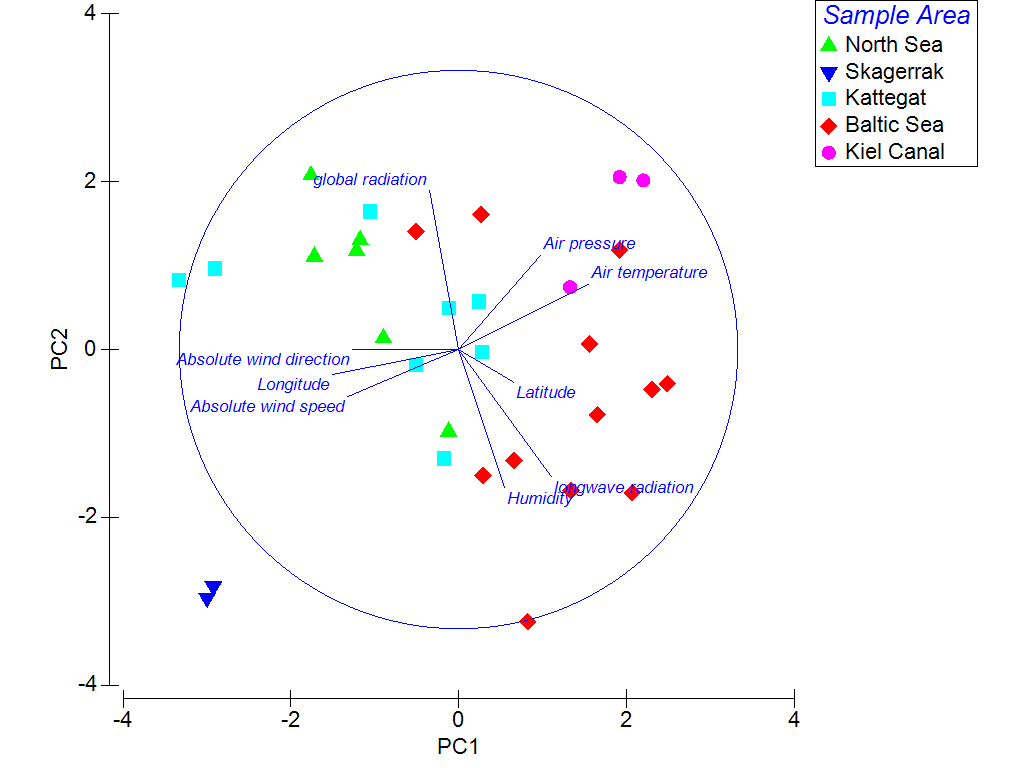


**Fig. S2** Principal components analysis (PCA) of the pair-wise distances of environmental variables measured for the samples calculated using the Euclidean distances.


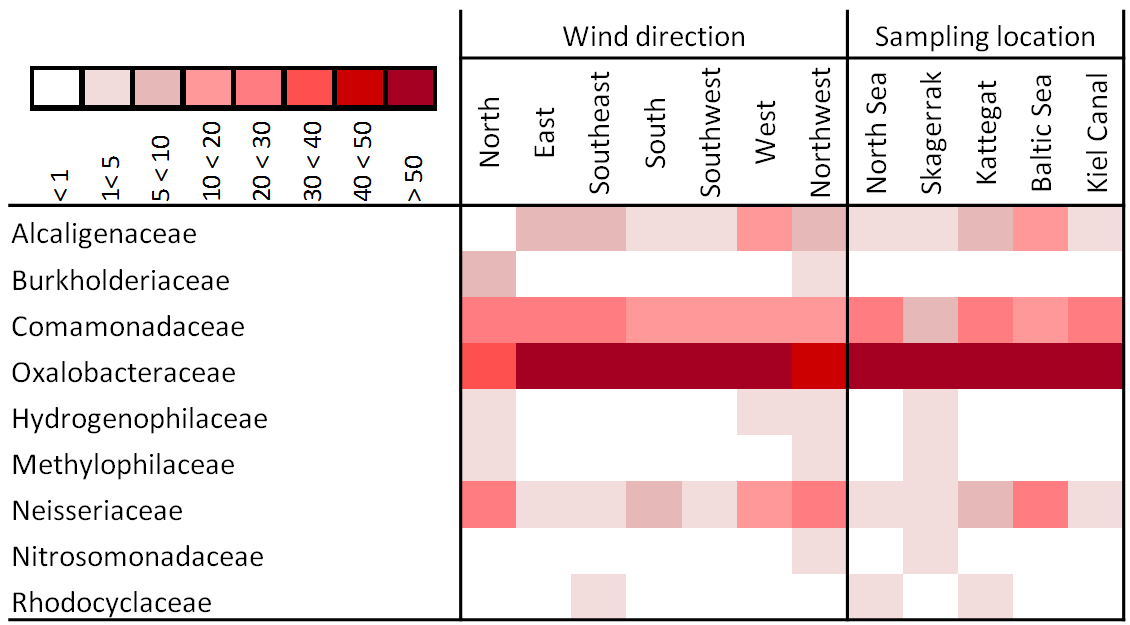


Fig. S3 Taxonomic classification of Betaproteobacteria reads grouped in wind direction and sampling location on family level using SILVA classifier based on 98% similarity omitting singletons (n = 1) and rare reads (< 1%). The amount of percentage proportion contribution of each family per group is indicated by color of cell; darker color represents higher contribution.


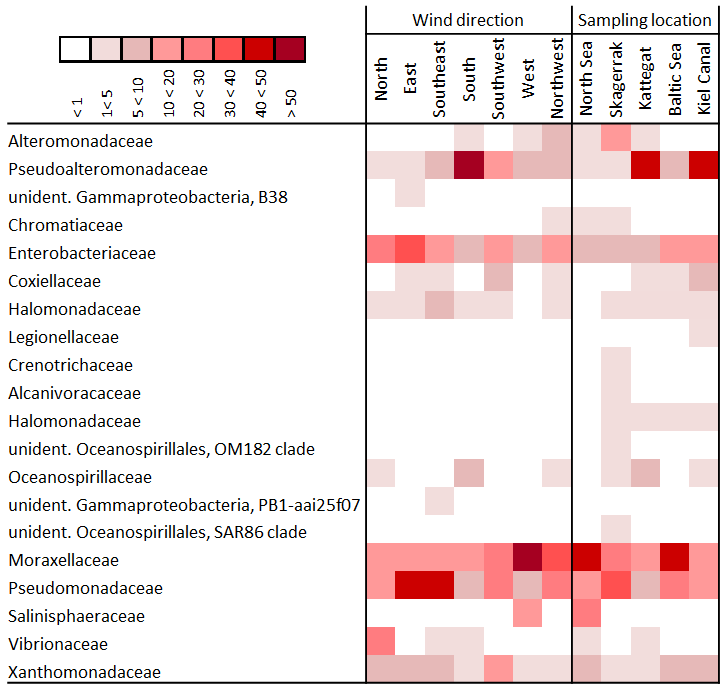


Fig. S4 Taxonomic classification of Gammaproteobacteria reads grouped in wind direction and sampling direction on family level using SILVA classifier based on 98% similarity omitting singletons (n = 1) and rare reads (< 1%). The amount of percentage proportion contribution of each family per group is indicated by color of cell; darker color represents higher contribution.


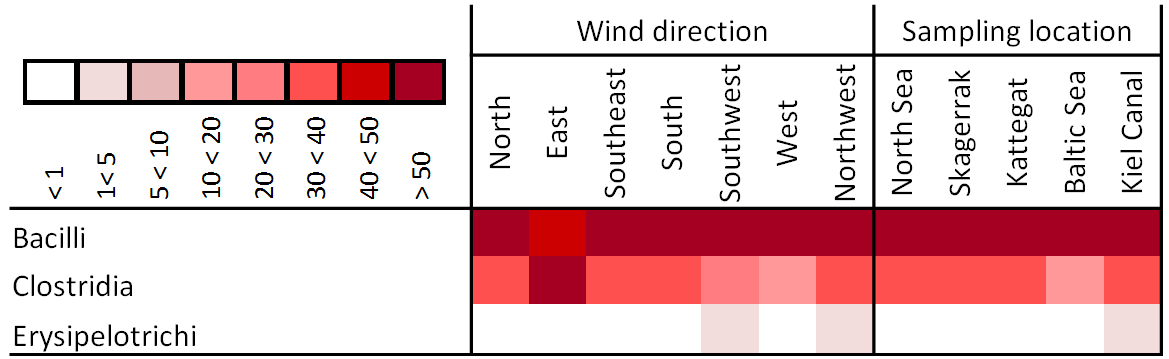


Fig. S5 Taxonomic classification of Firmicutes reads grouped in wind direction and sampling location on class level using SILVA classifier based on 98% similarity omitting singletons (n = 1) and rare reads (< 1%). The amount of percentage proportion contribution of each class per group is indicated by color of cell; darker color represents higher contribution.


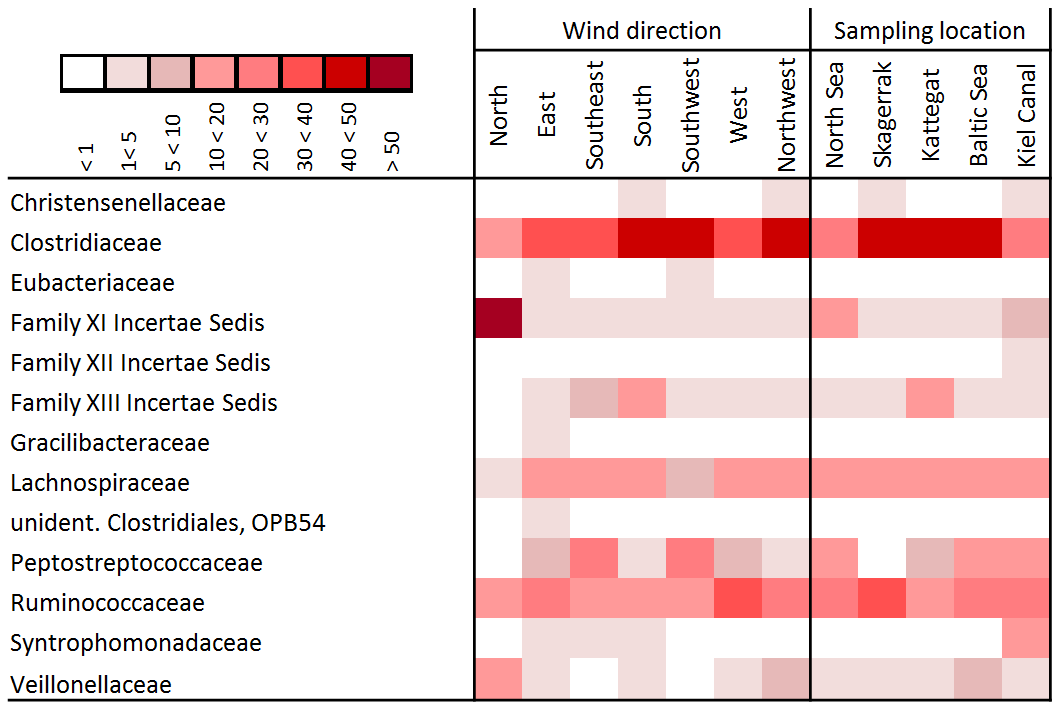


**Fig. S6** Taxonomic classification of Clostridia reads grouped in wind direction and sampling location on family level using SILVA classifier based on 98% similarity omitting singletons (n = 1) and rare reads (< 1%). The amount of percentage proportion contribution of each family per group is indicated by color of cell; darker color represents higher contribution.


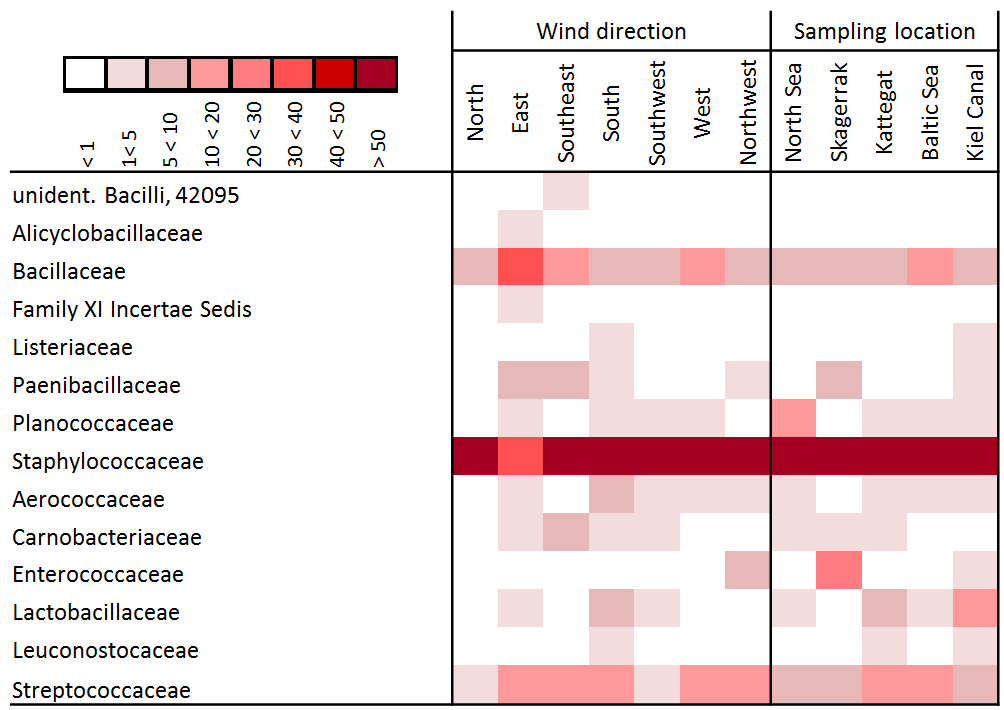


Fig. S7 Taxonomic classification of Bacilli reads grouped in wind direction and sampling location on family level using SILVA classifier based on 98% similarity omitting singletons (n = 1) and rare reads (< 1%). The amount of percentage proportion contribution of each family per group is indicated by color of cell; darker color represents higher contribution.


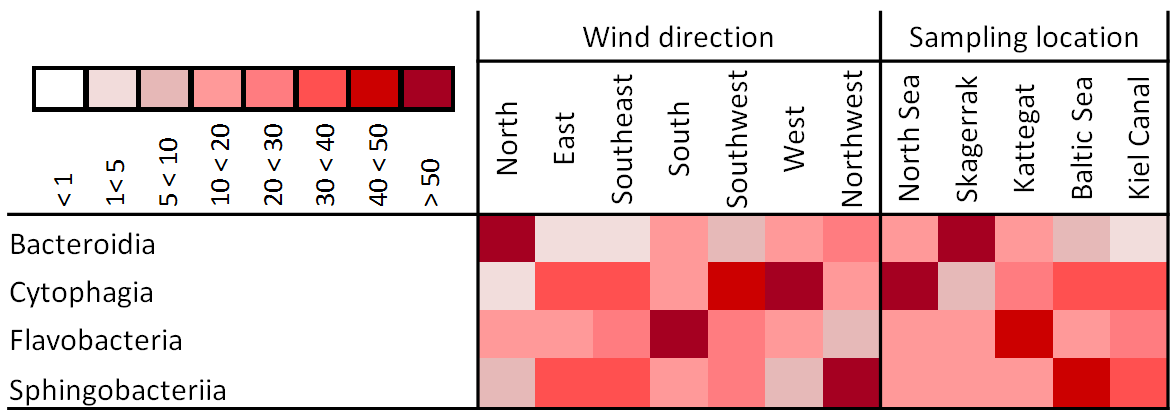


Fig. S8 Taxonomic classification of Bacteroidetes reads grouped in wind direction and sampling location on class level using SILVA classifier based on 98% similarity omitting singletons (n = 1) and rare reads (< 1%). The amount of percentage proportion contribution of each class per group is indicated by color of cell; darker color represents higher contribution.


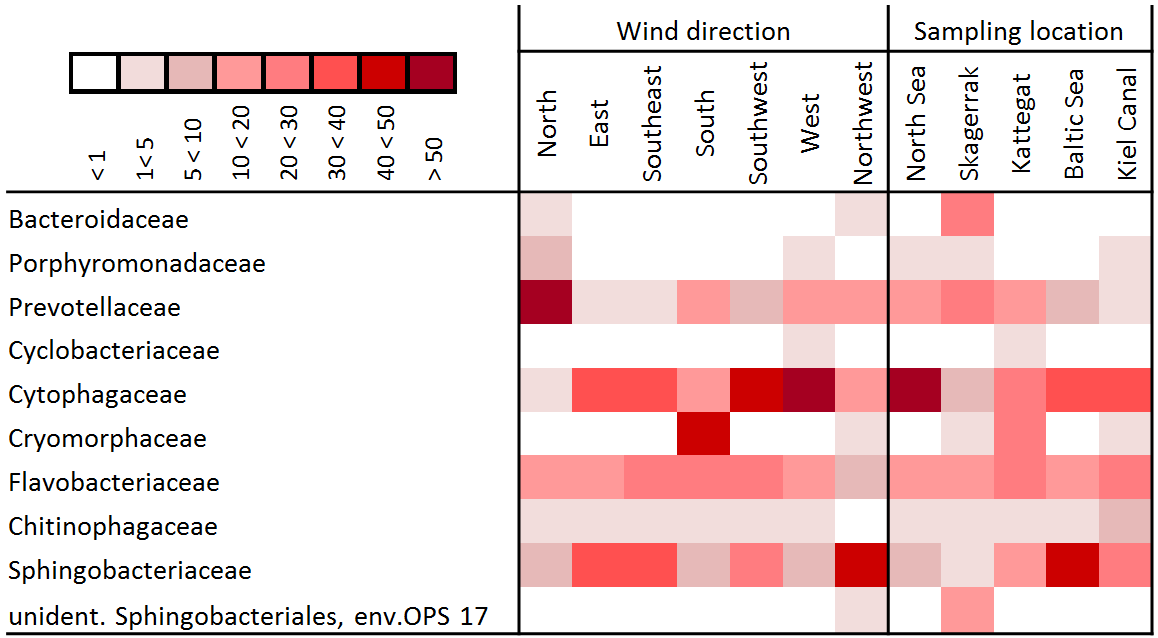


Fig. S9 Taxonomic classification of Bacteroidetes reads grouped in wind direction and sampling location on family level using SILVA classifier based on 98% similarity omitting singletons (n = 1) and rare reads (< 1%). The amount of percentage proportion contribution of each family per group is indicated by color of cell; darker color represents higher contribution.
