# Supplementary material for: Spatial distribution of marine airborne bacterial communities
Source: Microbiologyopen. 2015 Mar 19;4(3):475–90. doi: 10.1002/mbo3.253 (PMC4475389; doi:10.1002/mbo3.253)
Supplement: Supplementary file 2 [file mbo30004-0475-sd2.doc]

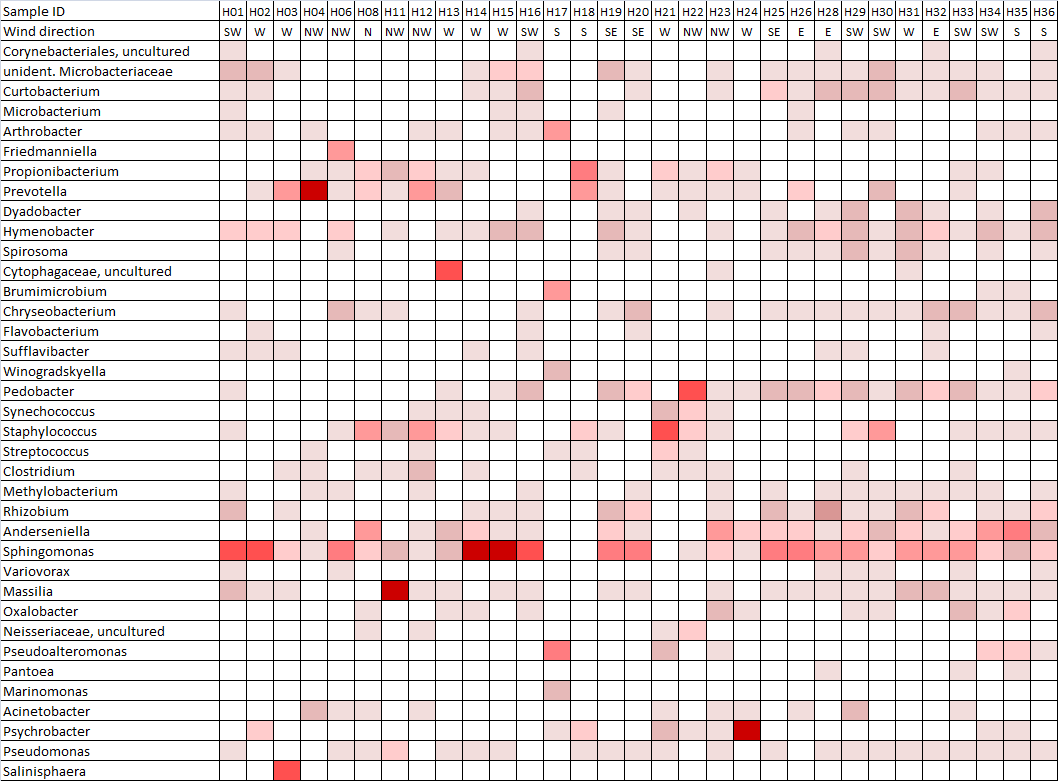


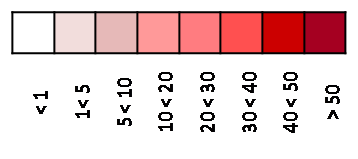


Fig.1: Relative distribution of the main taxa across the sampling locations and prevailing wind directions using SILVA classifier based on 98% similarity omitting singletons (n = 1) and rare reads (< 1%). The amount of percentage proportion contribution of each phyla per group is indicated by color of cell; darker color represent higher contribution.
